# Freeze-Dried CS/PVP/PVA Composite Scaffolds Doped with Curcumin and SiO_2_ Nanoparticles for Tissue Regeneration

**DOI:** 10.3390/ijms27156856

**Published:** 2026-07-30

**Authors:** Domingo Cesar Carrascal-Hernández, Jairo Ortiz, Alexander Córdoba, Paula A. Zapata, Carlos Humberto Valencia-Llano, Diego López-Tenorio, Diana Paola Navia-Porras, Johannes Delgado-Ospina, Eliceo Cortes, Juan David Rodríguez Macías, Carlos David Grande-Tovar

**Affiliations:** 1 Grupo de Investigación en Sociedad, Educación y Desarrollo Humano (GISEDH), Facultad de Ciencias, Educación, Artes y Humanidades, Institución Universitaria de Barranquilla, Barranquilla 080002, Colombia; 2 Grupo de Investigación de Fotoquímica y Fotobiología, Programa de Química, Universidad del Atlántico, Puerto Colombia 081007, Colombia; jairoaortiz@mail.uniatlantico.edu.co; 3 Grupo Polímeros, Departamento de Ciencias del Ambiente, Facultad de Química y Biología, Universidad de Santiago de Chile (USACH), Santiago 9170022, Chile; alexander.cordoba@usach.cl (A.C.); paula.zapata@usach.cl (P.A.Z.); 4 Grupo Biomateriales Dentales, Escuela de Odontología, Universidad del Valle, Cali 76001, Colombia; carlos.humberto.valencia@correounivalle.edu.co (C.H.V.-L.); diego.lopez.tenorio@correounivalle.edu.co (D.L.-T.); 5 Grupo de Investigación Biotecnología, Facultad de Ingeniería, Universidad de San Buenaventura Cali, Cali 76001, Colombia; dpnavia@usbcali.edu.co (D.P.N.-P.); jdelgado1@usbcali.edu.co (J.D.-O.); 6 Programa de Medicina, Facultad de Ciencias de la Salud, Exactas y Naturales, Universidad Libre, Barranquilla 081007, Colombia; juand.rodriguezm@unilibre.edu.co

**Keywords:** chitosan, curcumin, silicon dioxide nanoparticles, freeze-dried scaffolds, tissue regeneration

## Abstract

In this study, three formulations (F1, F2, and F3) were developed to fabricate hybrid scaffolds composed of chitosan (CS), polyvinylpyrrolidone (PVP), and polyvinyl alcohol (PVA), doped with curcumin (CUR) and silicon dioxide nanoparticles (NPs-SiO_2_). Structural analysis of the scaffolds was performed using Fourier transform infrared spectroscopy (FTIR), X-ray diffraction (XRD), thermogravimetric analysis (TGA), and scanning electron microscopy (SEM). This analysis demonstrated that incorporating bioactive molecules, such as curcumin (CUR) and silicon dioxide nanoparticles (NPs-SiO_2_), modified intermolecular interactions within the prepared scaffolds, thereby favoring the formation of more stable amorphous structures. This effect was most evident in scaffold formulation 3, which exhibited a more porous architecture with a uniform distribution of NPs-SiO_2_. Furthermore, mechanical evaluation of the formulations revealed that F2 exhibited the highest elastic modulus (0.773 ± 0.177 MPa) and compressive strength (1.015 ± 0.012 MPa), while F3 showed a more balanced combination of flexibility, structural stability, and displacement capacity. All formulations achieved complete inhibition of the Gram-positive and Gram-negative bacterial strains evaluated under the same test conditions. In vitro studies demonstrated that F3 maintained cell viability above 80% in normal BHK-21 fibroblasts while exhibiting enhanced cytotoxicity toward HEp-2 tumor cells (IC_50_ = 373.6 µg/mL). Additionally, subdermal implantation revealed the presence of trabeculae-like structures, osteocyte-like lacunae, and osteoblastic-like cells in F3, suggesting the initiation of osteogenic-type tissue organization. These findings identify F3 as the formulation with the most favorable balance of structural, mechanical, antimicrobial, and biological properties, supporting its potential application as a multifunctional scaffold for tissue regeneration.

## 1. Introduction

The loss of structural and functional integrity of tissues remains a major clinical challenge in regenerative medicine. Among these conditions, musculoskeletal injuries, particularly skeletal muscle damage, represent a significant proportion of cases associated with pain, functional impairment, fibrosis, and incomplete regeneration [1,2]. There is a wide spectrum of musculoskeletal disorders and injuries in general, caused by various conditions, that lead to morbidity in patients [3]. In fact, musculoskeletal conditions are the leading cause of disability in more than 160 countries worldwide. Furthermore, according to the World Health Organization (WHO), more than 1.71 billion people worldwide suffer from musculoskeletal conditions, which encompass more than 150 diseases [3].

Clinically, the management of musculoskeletal injuries (including soft tissue injuries) comprises a set of conventional therapeutic strategies, including surgical, physical, and pharmacological interventions, all of which present limitations and challenges [4,5,6]. Current surgical interventions are highly invasive, generate prolonged pain, and cause fibrosis in previously healthy tissues. While pharmacological interventions, such as NSAIDs, analgesics, and corticosteroids, are effective for symptomatic control (pain and inflammation), they can have serious side effects, including kidney damage and liver problems. Physical interventions, such as physiotherapy, are very slow and provide only limited recovery of mobility [7].

Several groundbreaking advances have been made in recent decades, facilitating the study and screening of a wide range of bioactive molecules, such as CUR. CUR has been used as a doping agent in nanostructured biomaterials due to its anti-inflammatory, antioxidant, immunomodulatory, and pro-regenerative properties. These properties are attractive because they promote favorable conditions in the tissue microenvironment; furthermore, they reduce fibrosis and oxidative stress associated with poor wound healing [8,9]. Doping lyophilized scaffolds with CUR is an interesting approach because it combines the compound’s biological activity with the structural advantages of porous, three-dimensional matrices. For example, several previous studies have shown that CUR-loaded scaffolds can improve cell adhesion and proliferation, enhance scaffold stability, and support regenerative processes by modulating local inflammatory responses [10]. Additionally, the synergistic interaction between polymer matrices and CUR has been associated with improved mechanical performance and microarchitectural characteristics that promote tissue regeneration, highlighting its potential as a multifunctional component in scaffold-based regenerative therapies [11].

In addition to CUR’s biological activity, the scaffold’s polymeric and inorganic components contribute complementary properties essential for tissue regeneration. Chitosan (CS) possesses fundamental properties due to the presence of amino groups (–NH_2_), which confer intrinsic antimicrobial activity, modulate early inflammatory responses, and contribute to the formation of porous networks that facilitate cell infiltration and extracellular matrix deposition [12]. In recent years, several studies have reported that CS-based biomaterials can promote osteogenic differentiation of mesenchymal stem cells, stimulate the formation of a mineralized matrix, and promote bone tissue regeneration through favorable cell–material interactions and modulation of the local regenerative microenvironment. These properties have positioned CS as an attractive biomaterial for applications requiring not only structural support but also active participation in tissue repair and osteogenic processes [13,14]. Polyvinylpyrrolidone (PVP) enhances scaffold hydration, colloidal stability, and the homogeneous distribution of bioactive compounds, thereby improving flexibility and compatibility with host tissues [15]. Polyvinyl alcohol (PVA), widely used in tissue engineering, offers excellent water solubility, biocompatibility, and controlled degradation, thereby forming stable three-dimensional networks that support cell adhesion and tissue organization [16]. In addition, silicon dioxide nanoparticles (NPs-SiO_2_) provide structural reinforcement through their high specific surface area and nanometric topography, while also promoting early cell–material interactions that may favor regenerative processes [17].

The combination of these materials in freeze-dried hybrid scaffolds enables the integration of structural support, biological activity, antimicrobial protection, and favorable physicochemical properties into a single multifunctional platform [18]. Several recent advances in regenerative medicine have highlighted the importance of multifunctional scaffolds capable of simultaneously providing structural support, biological stimulation, antimicrobial protection, and controlled release of bioactive molecules. For example, Cheng et al. reported significant recent advances in the design of electrospinning-fabricated multifunctional platforms, which have demonstrated a range of medical applications, including the delivery of drugs, stem cells, growth factors, antibacterial and anti-inflammatory agents, hemostasis, wound healing, and tissue repair and regeneration [19]. Furthermore, the development of enzyme-doped hydrogel-based materials printed by digital light processing has enabled spatiotemporal immunomodulation and the repair of infected wounds, as in the study reported by Cheng et al., which integrated ZIF-8 enzymes, tannic acid, and MXene into a gelatin methacrylate (GelMA) and CS methacrylate (CSMA) (ZGC) matrix for controlled wound treatment applications [20].

Additionally, polymeric systems containing silica-based nanomaterials have been reported to influence cell behavior and extracellular matrix organization, suggesting the potential to induce regenerative responses beyond simple tissue replacement [21]. Despite the extensive use of polymeric scaffolds based on CS, PVA, and PVP, limited information is available regarding the combined incorporation of curcumin and NPs-SiO_2_ into freeze-dried hybrid scaffolds and their influence on structural, mechanical, antimicrobial, and biological performance. Therefore, this study aimed to develop freeze-dried CS/PVP/PVA scaffolds doped with CUR and NPs-SiO_2_ and evaluate their structural, thermal, mechanical, antimicrobial, in vitro, and in vivo biological performance as bioactive platforms for tissue regeneration.

## 2. Results and Discussion

### 2.1. FTIR Characterization of CS/PVP/PVA/CUR/NPs-SiO_2_ Scaffolds

**Figure 1:**
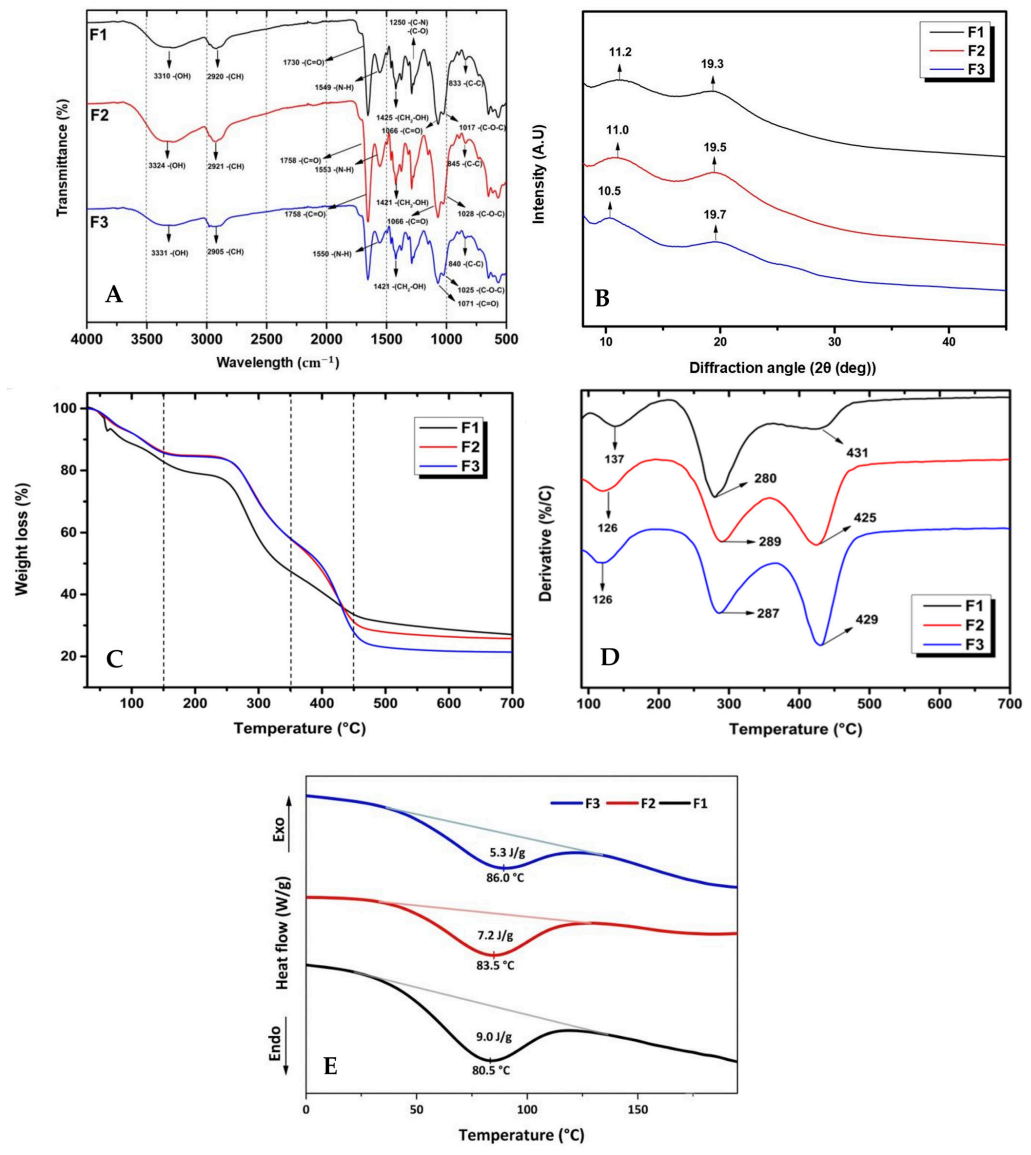
CS/PVP/PVA/CUR/NPs-SiO_2_ scaffold characterization by (**A**) FT-IR, (**B**) X-ray diffractometry (XRD), (**C**) thermogravimetric analysis (TGA), (**D**) derivative of TGA (DTGA), (**E**) differential scanning calorimetry (DSC). F1: Black; F2: Red; F3: Blue. F1: CS40%/PVP40%/PVA20%. F2: CS40%/PVP40%/PVA18%/CUR1%/NPs-SiO_2_1%. F3: CS40%/PVP40%/PVA16%/CUR 2%/NPs-SiO_2_ 2%. The FTIR and the TGA thermogram were reproduced from [26], licensed under CC BY 4.0.

The FTIR spectra (Figure 1A) confirmed the formation of a hybrid polymeric network composed of CS, PVP, and PVA and showed that the incorporation of CUR and NPs-SiO_2_-modified intermolecular interactions without altering the fundamental structure of the scaffolds [22]. Shifts observed in the hydroxyl and carbonyl regions indicate a reorganization of the hydrogen-bonding network following the incorporation of the bioactive components [23]. The preservation of the characteristic bands associated with amino, hydroxyl, and carbonyl groups indicates that the structural integrity of the polymer matrix was maintained. In addition, the increased intensity of bands in the low-wavenumber region and around 1240–1270 cm^−1^ supports the successful incorporation of NPs-SiO_2_ and CUR into the scaffold formulations [24]. Overall, the FTIR results indicate that the additives were integrated into the polymeric matrix through physicochemical interactions that favor the formation of a stable hybrid structure [25].

### 2.2. XRD Analysis of the Scaffolds

The XRD patterns of all formulations (Figure 1B) displayed broad diffraction bands characteristic of predominantly amorphous polymeric systems [27]. Compared with F1, formulations containing CUR and NPs-SiO_2_ exhibited reduced peak intensity and slight shifts in diffraction maxima, indicating a progressive decrease in crystallinity and increased structural disorder [28]. These changes suggest strong interactions among CS, PVA, PVP, CUR, and silica nanoparticles, which limit molecular rearrangement and favor the formation of a homogeneous hybrid network [29]. The predominance of amorphous domains may facilitate fluid penetration, mass transport, and molecular diffusion within the scaffold matrix, characteristics that are desirable for tissue engineering applications [30].

From a functional perspective, the formation of predominantly amorphous matrices, as suggested by the diffractograms of F1, F2, and F3, may be advantageous for future controlled delivery applications. The elimination of rigid crystalline regions may facilitate molecular diffusion within the matrix and could be beneficial for future controlled delivery applications. Furthermore, the amorphous dispersion of curcumin within the polymer matrix may improve its apparent distribution and potentially favor its availability in biological environments [31].

### 2.3. Thermal Gravimetric Analysis (TGA) and Differential Scanning Calorimetry (DSC) of CS/PVP/PVA/CUR/NPs-SiO_2_ Scaffolds

TGA and DTGA (Figure 1C,D) analysis demonstrated a multistage degradation behavior characteristic of hybrid polymeric systems. All formulations exhibited an initial mass loss associated with water removal, followed by decomposition of the polymeric network and the formation of thermally stable residues at higher temperatures. Formulations containing CUR and NPs-SiO_2_ exhibited improved thermal stability compared with F1, indicating stronger intermolecular interactions and a stabilizing effect from the inorganic phase [32].

The DSC thermograms (Figure 1E) revealed a progressive increase in glass transition temperature from F1 to F3, accompanied by a reduction in transition enthalpy [33]. These results indicate reduced chain mobility and the formation of a more compact and homogeneous network after incorporation of CUR and NPs-SiO_2_ [34]. The thermal analysis suggests that the incorporation of CUR and NPs-SiO_2_ contributes to the development of structurally integrated scaffolds with enhanced thermal resistance [35].

From a structural perspective, this dual effect suggests that the system transitions from a flexible, energetically active network (as in F1) to a more compact, restricted, and homogeneous matrix (as in F3). Furthermore, the presence of NPs-SiO_2_, along with the interaction of CUR with polymers, may introduce additional polymer–nanoparticle interaction sites and strengthen intermolecular interactions, thereby limiting mobility between segments [36]. Consequently, the fraction of thermally active domains that can contribute to the endothermic event decreases, thereby reducing the enthalpy [37].

### 2.4. Scanning Electron Microscopy (SEM) and SEM-EDS of CS/PVP/PVA/CUR/NPs-SiO_2_ Scaffolds

**Figure 2:**
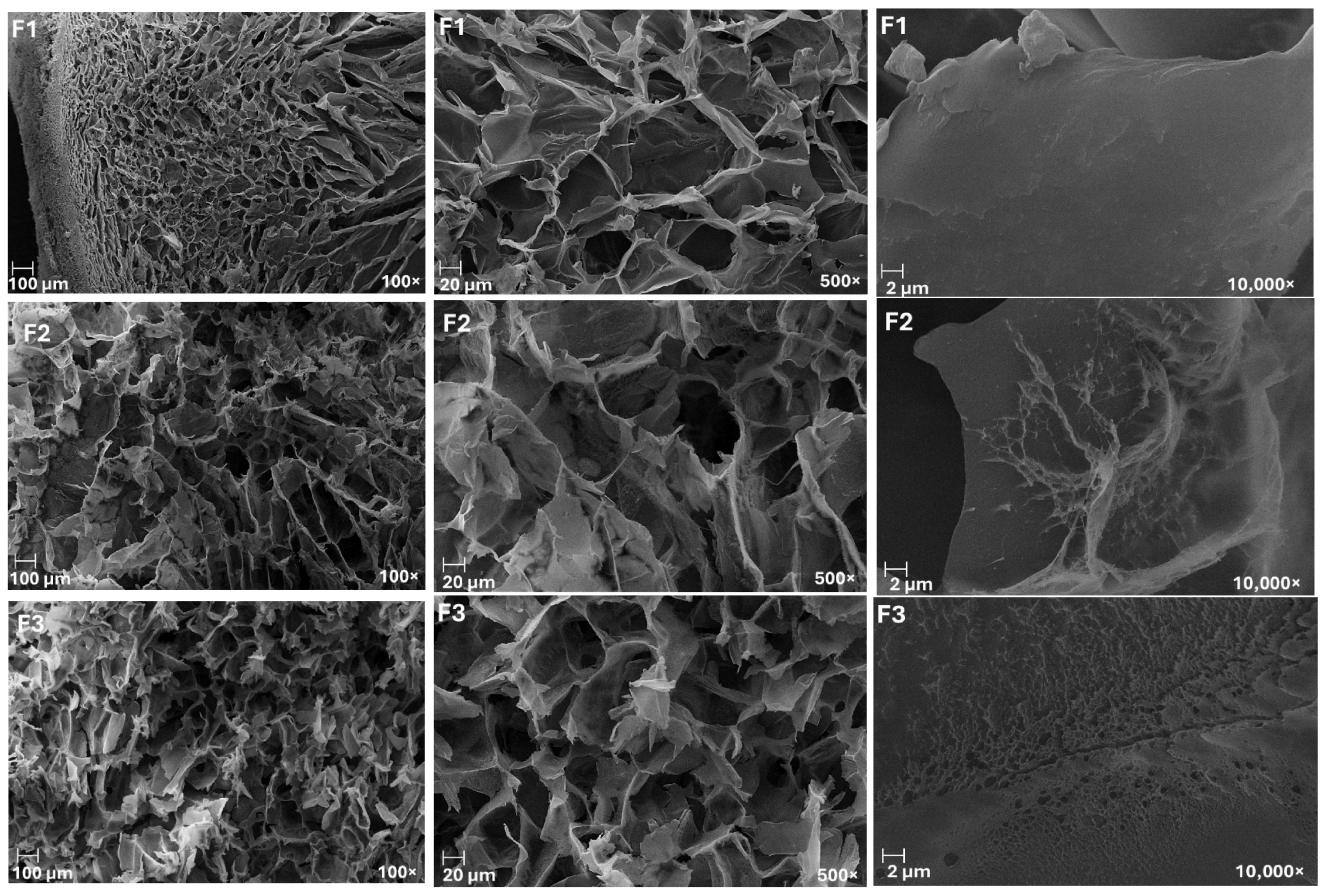
Micrograph of the CS/PVP/PVA/CUR/NPs-SiO_2_ scaffolds: F1: CS40%/PVP40%/PVA20%. F2: CS40%/PVP40%/PVA18%/CUR1%/NPs-SiO_2_1%. F3: CS40%/PVP40%/PVA16%/CUR 2%/NP-SiO_2_ 2%.

The micrographs in Figure 2 show the morphology of formulations F1, F2, and F3 at magnifications of 100×, 500×, and 10,000×. These images demonstrate that the scaffold’s final architecture is directly determined by the chemical interactions that occur during its synthesis and freeze-drying. In F1, the absence of nanoparticles indicates a system governed solely by hydrogen bonding among CS, PVP, and PVA. Although present, these bonds are insufficient to balance the internal stresses generated by the nucleation and growth of water ice crystals and by residual acetic acid during polymer synthesis [38]. This explains the presence of large, collapsed, and irregular pores, as well as wavy and microfolded walls, resulting in a heterogeneous structure that reflects a polymer network without stable anchor points and exhibiting high fragility [39,40]. By incorporating NPs-SiO_2_ into F2, a characteristic morphology is evident that suggests the entry into a new chemical regime: the NPs-SiO_2_ may contribute to the stabilization of the porous architecture through interactions previously suggested by FTIR analysis between CS, PVP, and PVA chains, reducing segmental mobility, stabilizing the scaffold structure, and conferring a more stable morphology, with more defined pores and fewer collapsed walls [40].

In F3, a greater consolidation of the material’s porosity is observed, where the higher concentration of NPs-SiO_2_ leads to a deeper reorganization of the system. Additionally, PVP plays a crucial role in this consolidation process: its highly hydrophilic rings and strong hydrogen-bonding capacity enhance the compatibility of the polymer chain and facilitate the dispersion of the NPs-SiO_2_ throughout the matrix [39]. The -C=O group of pyrrolidone establishes secondary interactions with both the polymer network and the silica surface, improving colloidal stability and reducing microphase separation. F3 exhibits a more suitable porous structure with uniform organization, influenced by synergistic interactions among the scaffold components (CS, PVA, PVP, and NPs-SiO_2_). These results suggest the formation of a more integrated hybrid scaffold internal structure in F3, where the inorganic phase not only stabilizes the architecture but also defines much of its thermal, mechanical, and morphological integrity. Thus, the F1-F2-F3 sequence not only describes changes in observable porosity but also the chemical evolution of a system that progresses from the inherent fragility of the pure polymer matrix to the regulated structure of a coherent and chemically organized composite.

The micrographs of the formulations presented in Figure 2 are complemented by scanning electron microscopy coupled with energy-dispersive X-ray spectroscopy (SEM-EDS) to confirm the incorporation of CUR and NPs-SiO_2_. The SEM micrographs in Figure 2 reveal a highly porous, three-dimensional architecture with irregular pores predominantly <50 µm. In this regard, F1 exhibits a continuous and homogeneous texture. Table 1 reports data for F1, with a mean pore diameter of 23.3 ± 31.3 µm and an elongation of 2.34 ± 1.18 µm, indicating a relatively stable morphology but with high variability (high standard deviation), consistent with the local heterogeneity typical of lyophilized matrices [41]. In the case of F2, rougher surfaces and shinier areas are evident, possibly due to nanoparticles embedded in the walls, thereby modifying the topography. Table 1 reports a thickness of 18.1 ± 22.9 µm and an elongation of 1.91 ± 0.78, values that reflect a reduction in the mean compared to the control and a lower mean elongation, along with greater variability. This is consistent with the effect of the additives. F3 shows a more homogeneous morphology, with thinner walls and a fine texture at high magnifications, suggesting better dispersion of the additives in the matrix, with values of 30.0 ± 29.6 µm and an elongation of 2.08 ± 0.78; that is, the highest mean value among the formulations, but with a high standard deviation. This suggests that, although the overall trend is toward a more open and resistant architecture, local variations persist between fields that must be considered in the functional interpretation.

The pore size and elongation index distributions showed positive skewness, a typical behavior of freeze-dried matrices characterized by the coexistence of micropores and macropores. The average elongation index values were 2.34 (F1), 1.91 (F2), and 2.08 (F3). Furthermore, given the non-normal distribution of the data, as evidenced by the Shapiro–Wilk test (*p* < 0.05), the results are presented as medians and interquartile ranges [IQRs]. This non-parametric approach provides a more accurate proportional representation of the predominant pore size because it is less sensitive to atypical macropores formed by the crystals. The data obtained were F1 = 1.917 [1.536–2.483] (2.336 ± 1.184); F2 = 1.711 [1.402–2.163] (1.906 ± 0.783); F3 = 1.891 [1.595–2.395] (2.084 ± 0.782), and the Kruskal–Wallis test indicated that there were no significant differences in elongation (H = 5.48; *p* = 0.064). Post hoc Dunn–Bonferroni comparisons confirmed the absence of statistical significance.

**Figure 3:**
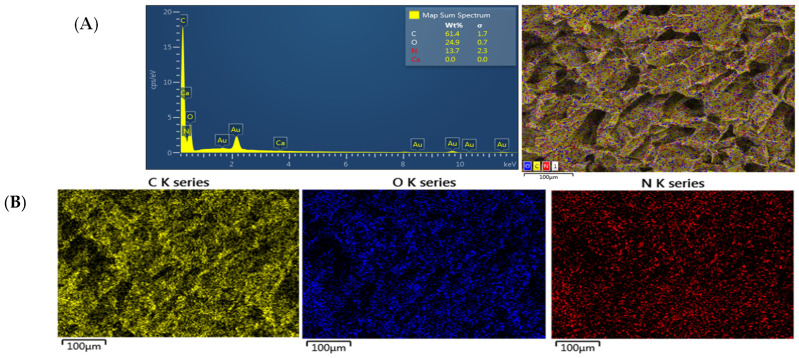
SEM-EDS analysis of formulation F1: CS 40%/PVP 40%/PVA 20%. (**A**) The characteristic peaks of C, O, and N and their respective weight percentages are observed. (**B**) Showing the spatial distribution of carbon (C K series), oxygen (O K series), and nitrogen (N K series), supporting the incorporation of SiO_2_ nanoparticles and the homogeneous distribution of the scaffold components (scale: 100 μm).

Figure 3 shows the SEM-EDS results for F1, which exhibit carbon (61.4 ± 1.7 wt%), oxygen (24.9 ± 0.7 wt%), and nitrogen (13.7 ± 2.3 wt%) percentages that closely match the theoretical compositions of the constituent polymers [42]. The carbon and oxygen originate from the CS polysaccharide backbone, as well as the abundance of C–C, C–O, and C=O bonds present in PVP and PVA [43]. The nitrogen is associated exclusively with the -NH_2_ groups of CS and the lactam ring of PVP, providing a chemically significant marker of its presence in the mixture. Minor signals corresponding to gold were detected, likely due to the spray coating (or metallization) applied to the sample to make it conductive before SEM-EDS observation (Figure 3A). It is important to note that no inorganic elements, such as Ca or Si, were detected, confirming that F1 is a purely polymeric matrix and validating its use as a reference scaffold for comparison with nanoparticle-loaded systems, as in F2 and F3. Comparable elemental profiles, dominated by C, O, and N, have been reported for PVA/PVP/CS blends, which are generally interpreted as evidence of compositional purity and effective polymer integration [43].

Figure 3B shows elemental mapping of the scaffold, further clarifying the internal chemical homogeneity of formulation F1. Carbon (C K series) exhibits continuous distribution throughout the analyzed surface, consistent with a chemically integrated organic matrix. Oxygen (O K series) follows a similar spatial pattern, with intensity modulations primarily associated with surface topography rather than compositional variations [42]. These observations indicate that oxygen-rich functional groups appear broadly distributed within the scaffold walls. Furthermore, the nitrogen mapping (N K series) shows a homogeneous signal with no evidence of localized enrichment or depletion. Since nitrogen is a selective indicator of CS and PVP, its spatial distribution suggests that these polymers remain well dispersed throughout the analyzed region at the micro scale [42]. In polymer blend systems, this type of elemental distribution is commonly associated with good compatibility among components, and no evidence of microscale phase separation was observed within the resolution limits of SEM-EDS [44]. Furthermore, the observed elemental uniformity contrasts sharply with polymer systems, where EDS mapping typically reveals distinct domains enriched in specific elements. The absence of such domains in F1 implies favorable intermolecular interactions, likely mediated by hydrogen bonds between –OH, –NH_2_, and C=O groups, which have been shown to promote compatibility in CS/PVP/PVA systems [44].

**Figure 4:**
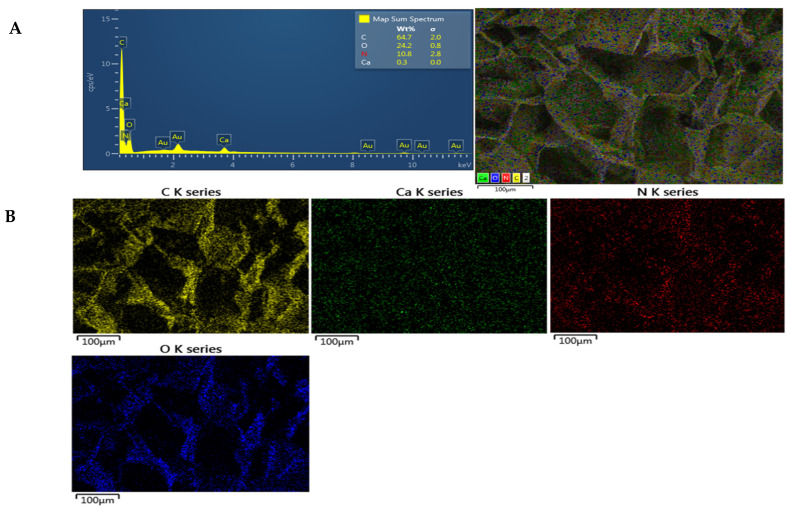
SEM-EDS analysis of formulation F2: CS40%/PVP40%/PVA18%/CUR1%/NPs-SiO_2_1%. (**A**) The presence of C, O, and N as the main elements of the polymer matrix is confirmed, as well as signals associated with the NPs-SiO_2_, while the Au peaks originate from the conductive coating. (**B**) The distribution of C, O, N, and Ca is shown, supporting the presence of NPs-SiO_2_ and the relatively uniform microscale distribution of the scaffold components within the analyzed area (scale: 100 μm).

Furthermore, the SEM-EDS micrograph of F2, shown in Figure 4, reveals notable differences compared to the base polymeric scaffold (F1), reflecting the structural and chemical impact of incorporating CUR and silica-based NPs into the CS/PVP/PVA matrix. The SEM-EDS micrographs in Figure 4A show that F2 retains the overall porous and interconnected architecture induced by the freeze-drying process; however, the pore walls appear slightly thicker and more irregular than those of F1 [29]. This change in the scaffold suggests that the introduction of CUR and inorganic NPs modifies the polymer–polymer and polymer–filler interactions during ice crystal growth and sublimation, leading to a more heterogeneous local microstructure. Despite these changes, the pores remain open and interconnected at the micrometer scale, indicating that functionalization does not compromise the material’s structural continuity [29].

Figure 4B shows that F2 is abundant in carbon (64.7 wt%), oxygen (24.2 wt%), and nitrogen (10.8 wt%), confirming that the polymer matrix remains the scaffold’s main structural component. Compared to F1, there is a relative decrease in nitrogen content, which may be associated with the lower proportion of nitrogen-rich polymer segments relative to the total scaffold mass after the incorporation of CUR and inorganic additives [43]. The consistent presence of nitrogen suggests that in the CS scaffold, the PVP remains well integrated into the scaffold matrix. Another interesting finding is the presence of calcium (approximately 0.3 wt.), which appears uniformly throughout the analyzed area. Although calcium is not a nominal component of the formulation, its low abundance and homogeneous distribution suggest that it is not present as a discrete crystalline phase, but rather as a trace element likely introduced during processing or handling [45].

**Figure 5:**
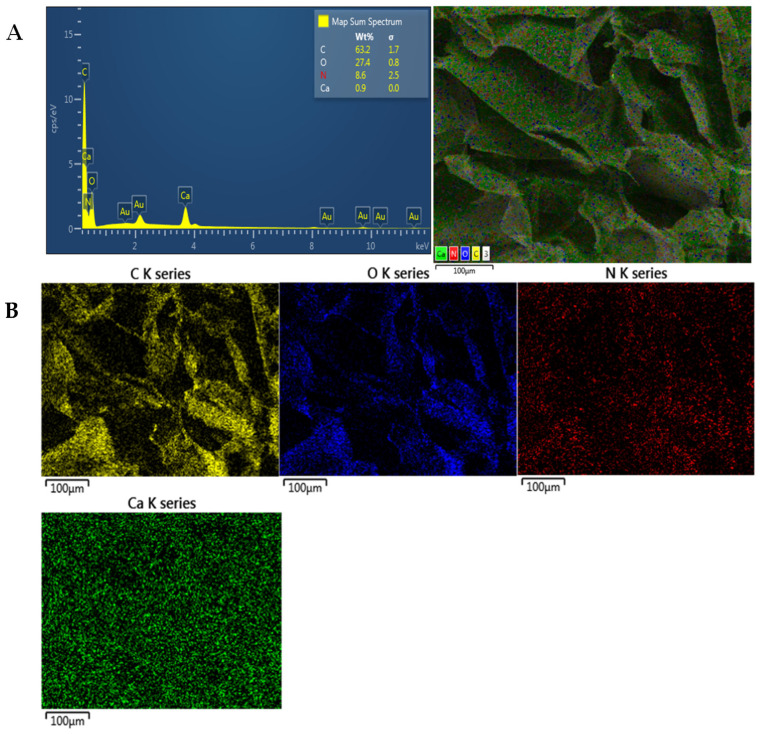
SEM-EDS analysis of formulation F3: CS 40%/PVP 40%/PVA 16%/CUR 2%/NP—SiO_2_ 2%. (**A**) confirms the presence of C, O, and N from the polymer matrix and CUR, as well as signals associated with NPs-SiO_2_, while the Au peaks originate from the conductive coating. (**B**) shows the distribution of C, O, N, and Ca, supporting the presence of NPs-SiO_2_ and the relatively uniform microscale distribution of the scaffold components in the analyzed region.

It is important to note that no localized calcium-rich domains are observed, ruling out the formation of segregated inorganic clusters at the micro scale. Elemental mapping further supports these observations. The carbon and oxygen maps show continuous and overlapping distributions, confirming that the organic matrix maintains chemical continuity along the scaffold walls (Figure 5). The nitrogen map reveals a broadly distributed signal with no evidence of pronounced elemental segregation. These observations suggest that major phase separation was not detected within the spatial resolution limits of the SEM-EDS technique [42]. This indicates that incorporating CUR and inorganic species does not alter the spatial distribution of nitrogen-containing polymers, suggesting that phase separation between the CS/PVP domains and the rest of the matrix does not occur at the SEM-EDS resolution [46].

In the case of formulation F3 (Figure 5A), clear evidence of cumulative structural and compositional effects induced by the higher loading of CUR and NPs-SiO_2_ within the CS/PVP/PVA matrix is observed. Morphologically, the scaffold retains the characteristic porous architecture of freeze-dried systems; however, unlike F1 and F2, the pore walls are noticeably thicker and more textured, with more defined edges and greater surface irregularity. These morphological changes suggest that the higher functional loading actively participates in the solidification process, altering the local viscosity and polymer rearrangement during freezing, resulting in a more compact and heterogeneous strut morphology [47].

Despite these changes, the porous network remains interconnected, and no collapsed regions or completely occluded pores are observed at the micrometer scale. This observation indicates that even at higher concentrations of CUR and inorganic species, the scaffold architecture remains structurally continuous, although it approaches a denser regime than the reference polymer system. EDS results in F3 indicate the presence of carbon (63.2 wt%) and oxygen (27.4 wt%), confirming that the organic polymer matrix continues to define the scaffold’s overall composition. Regarding nitrogen, the study reports a lower content (8.6 wt%) than in F1 and F2, reflecting a progressive dilution of nitrogen-containing polymer segments as the proportion of CUR and inorganic components increases. This result is consistent with a redistribution within a still-continuous matrix, in which nitrogen-rich domains remain present but constitute a smaller fraction of the total scaffold mass.

Although calcium was not intentionally added to the formulations, low-intensity Ca signals were consistently detected in F2 and F3. The origin of this element cannot be conclusively determined from SEM-EDS analysis alone; however, its homogeneous distribution suggests that it is present as a trace component rather than as a segregated mineral phase.

Elemental maps provide additional information on the internal chemical organization of F3. Carbon and oxygen distributions remain continuous along the scaffold walls, supporting the persistence of a chemically connected organic matrix (Figure 5B). The nitrogen map, although less intense, shows a broad, uniform distribution, indicating that the CS- and PVP-derived fractions remain spatially integrated within the scaffold. The absence of low-nitrogen regions or clearly defined boundaries contradicts the concept of microscale phase separation, even with this higher degree of functionalization.

SEM-EDS results indicate that F3 is a highly functionalized hybrid scaffold in which the polymer matrix, CUR, and inorganic species coexist within a single chemically integrated structure. Compared with F2, F3 exhibits a more pronounced modification of the pore wall morphology and a clearer inorganic fingerprint, while maintaining a relatively uniform elemental distribution at the micro scale, according to SEM-EDS analysis. This balance between increased functional content and maintained structural coherence highlights F3 as the upper limit of functionalization within the current formulation strategy, providing a valuable platform for exploring trade-offs between bioactive loading, structural integrity, and performance in subsequent physicochemical and biological evaluations.

### 2.5. Mechanical Properties of CS/PVP/PVA/CUR/NPs-SiO_2_ Scaffolds

In regenerative medicine, the use of potential biomaterials for implants requires rigorous analysis of their mechanical properties, a fundamental aspect for determining their suitability for regenerative applications [48]. In this regard, it is possible to design various formulations with specific mechanical properties that resemble those of natural tissue and promote tissue regeneration. Furthermore, they can be chemically modified to improve their biocompatibility, degradability, and response to biochemical signals from the physiological microenvironment [35]. In our study, we evaluated three formulations (F1, F2, and F3), whose mechanical test results are reported in Table 2.

The crystallinity of these formulations is shown in Figure 1B (X-ray diffraction analysis), which highlights factors influencing their mechanical properties, including the presence of amorphous regions. The predominance of amorphous regions may influence chain mobility and stress dissipation mechanisms, thereby contributing to the flexibility observed in these scaffolds [49].

All formulations exhibited the mechanical characteristics of flexible polymeric scaffolds, including relatively low elastic modulus values and high deformation capacity (Table 2). Among them, F2 presented the highest elastic modulus, compressive strength, and maximum force, indicating more efficient reinforcement of the polymer matrix. In contrast, F3 exhibited a more balanced combination of flexibility, displacement capacity, and structural stability, while maintaining mechanical resistance comparable to F1 [35].

These findings demonstrate that incorporating CUR and NPs-SiO_2_ significantly influences scaffold mechanics and that the final mechanical response depends on the balance between polymer mobility and filler-induced reinforcement [50].

### 2.6. Antimicrobial and Antibiofilm Evaluation of CUR and NPs-SiO_2_

Antimicrobial evaluation was performed on the isolated components (CUR and NPs-SiO_2_) to better understand their potential contribution to the biological behavior of the hybrid scaffolds. Three Gram-negative bacterial strains (*Enterobacter hormaechei*, *Klebsiella variicola*, and *Salmonella enterica*) and one Gram-positive strain (*Staphylococcus aureus*) were used. These strains are associated with serious opportunistic infections, a high capacity for biofilm formation, persistence in hospital environments, and, in some cases, multidrug resistance to antibiotics [51,52]. For example, *S. aureus* is one of the main etiological agents of skin, soft tissue, and surgical wound infections and harbors clinically relevant resistant variants [51]. Therefore, selecting these pathogens is important because they are prevalent in biomedical implantation settings, wounds, and surgical procedures, where microbial contamination can compromise material integration and tissue regeneration.

The antimicrobial evaluation of the NPs-SiO_2_ showed no significant bactericidal activity against the tested strains within the experimental range (Table 3). All minimum inhibitory concentration (MIC) values were reported as greater than 60 mg/mL, indicating that even at the highest concentrations tested, no inhibitory effect was observed against the microorganisms. Since no measurable bactericidal effect was detected within the tested concentration range, minimum bactericidal concentration values (MBC) for NPs-SiO_2_ could not be determined [52]. This pattern was consistent in both Gram-negative bacteria (*E. hormaechei*, *K. variicola*, and *S. enterica*) and Gram-positive bacteria (*S. aureus*), ruling out cell wall-dependent selectivity and suggesting limited direct antimicrobial activity under the evaluated conditions.

On the other hand, CUR exhibited definite antimicrobial activity, although it was species-dependent. The MIC and MBC values reveal a clear susceptibility gradient. Specifically, *S. aureus* was the most sensitive strain, while *K. variicola* showed the greatest resistance, with *E. hormaechei* and *S. enterica* exhibiting intermediate levels of susceptibility. This distribution reinforces the previously observed trend in inhibition levels, in which Gram-negative bacteria generally exhibit lower susceptibility [53] and in which CUR can interfere with community organization processes at concentrations higher than those strictly necessary to affect cell viability [54].

Additionally, the ability of NPs-SiO_2_ and CUR to modulate Biofilm production in bacteria was evaluated. The test was carried out with concentrations lower than the MIC (Sub-MIC) (Table 3), which, instead of compromising viability, act as cellular stress signals.

The nanoparticles exhibited differential effects in modulating biofilms. The NPs-SiO_2_ exhibited consistent inhibition across all evaluated strains (42.45–72.51%), suggesting a stable physicochemical mechanism that hinders bacterial adhesion in both Gram-negative and Gram-positive strains, including *S. aureus*. On the contrary, CUR exhibited greater variability across species. While in *S. aureus* and *S. enterica*, inhibitions greater than 80% were achieved, in *E. hormaechei*, a stimulus to biofilm formation was observed. This phenomenon suggests that the stress caused by CUR could activate adaptive response mechanisms in *E. hormaechei*, as has been reported previously for biofilm-forming bacteria exposed to subinhibitory concentrations of antimicrobial compounds, which induce cell aggregation through the overexpression of secretion systems or adhesins [55,56].

Although CUR exhibits antimicrobial activity, the high concentrations required, especially against Gram-negative bacteria, limit its viability as a single agent. This positions NPs-SiO_2_ not as an active antimicrobial agent but as a support matrix that could modulate virulence-associated processes, such as biofilm formation, thereby favoring the formation of a nanostructured platform with functional potential for delivery systems or combined strategies [57]. In this sense, encapsulating CUR and NPs-SiO_2_ within polymeric scaffolds may be an interesting strategy to improve the performance of antimicrobial biomaterials.

### 2.7. Antimicrobial Evaluation of PVP/PVA/CS/NPs-SiO_2_ Scaffolds

It was determined that all three scaffold formulations inhibited detectable bacterial growth under the experimental conditions used. This finding indicates a strong antimicrobial effect against both Gram-positive and Gram-negative microorganisms. However, it is important to clarify that the methodology used in this study does not allow for complete differentiation between bacterial elimination, growth inhibition, bacterial retention on the scaffold surface, or other surface-related effects. Therefore, the results should be interpreted as evidence of antimicrobial efficacy under the evaluated conditions, rather than as direct proof of a specific antimicrobial mechanism. This performance confirms broad-spectrum antibacterial activity, as both Gram-negative and Gram-positive bacteria were completely inhibited, demonstrating the high efficacy of the designed scaffolds, their potential for biomedical applications requiring microbial control, and suggesting that the mechanisms of action are not derived from a single component, but rather from the chemical and topological synergy generated within the CS/PVP/PVA/CUR/NPs-SiO_2_ hybrid matrix.

All three scaffold formulations inhibited bacterial growth under the experimental conditions used. This finding suggests a strong antimicrobial effect against both Gram-positive and Gram-negative microorganisms [58]. In previous work, the ability of chitosan scaffolds to inhibit bacteria was demonstrated by Viloria Angarita et al. (2024) [59]. However, the methodology used in this study does not allow for complete differentiation between bacterial elimination, growth inhibition, bacterial retention on the scaffold surface, or other surface-related effects. Therefore, the results should be interpreted as evidence of antimicrobial efficacy under the evaluated conditions, rather than as direct proof of a specific antimicrobial mechanism. However, prominent research that explores these results in greater depth is cited [60,61,62]. For example, in *S. aureus*, where the cell wall is thicker (peptidoglycan layer), chitosan primarily induces microdisorganization of the murein layer, thereby facilitating the penetration of other scaffolding agents [63].

Although PVP and PVA do not possess direct antimicrobial activity, they do determine the physicochemical behavior of the scaffold, favoring surface moisture retention, which facilitates the mobility of active species (ions, radicals, curcumin), conferring colloidal stability to the system, preventing aggregation, and allowing for a homogeneous distribution of the active components, and providing adjusted crystallinity, which regulates diffusion into the bacterial microenvironment. In this sense, their function is essentially structural: they create a hydrophilic, flexible medium with moderate roughness, conditions that enhance the interaction between the matrix and the cell membrane, thereby amplifying the effects of CS and nanoparticles [64].

Based on previous reports in the literature, the antimicrobial performance observed in the present study may be associated with several complementary mechanisms contributed by the scaffold components [64,65]. However, these mechanisms were not directly investigated here and should therefore be regarded as plausible interpretations rather than experimentally confirmed pathways.

Although the scaffolds exhibited complete inhibition of detectable bacterial growth under the conditions evaluated, further studies are required to determine the relative contributions of bactericidal activity, bacteriostatic effects, bacterial adsorption, membrane disruption, and metabolic inhibition. Techniques such as bacterial adhesion assays and membrane integrity analyses would provide a more comprehensive mechanistic understanding of the antimicrobial behavior of these hybrid scaffolds [65].

### 2.8. Study of the In Vitro Cellular Biocompatibility of the Scaffold

**Figure 6:**
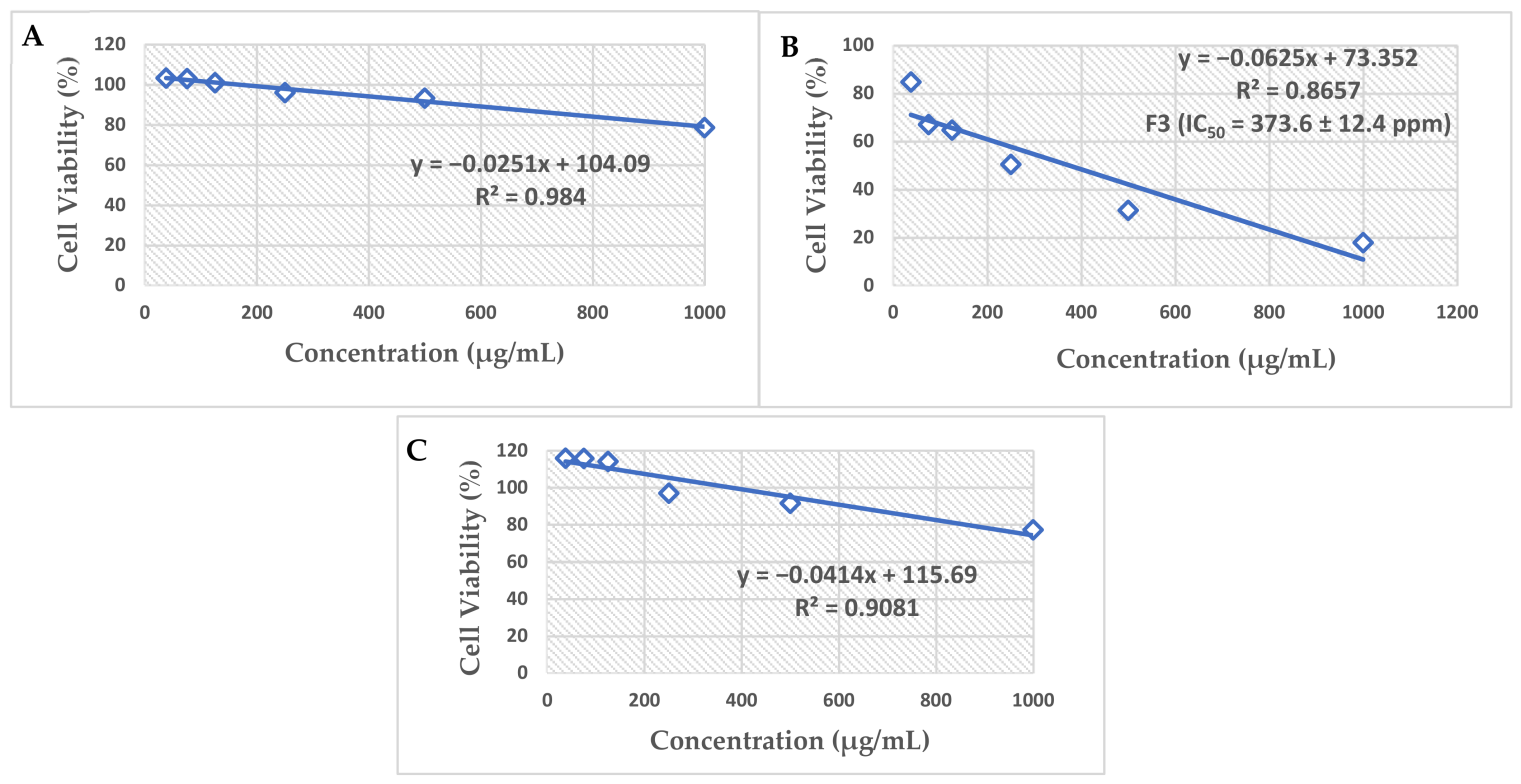
Dose–response curves of formulations F1 (**A**), F2 (**B**), and F3 (**C**) in HEp-2 cells after exposure to concentrations ranging from 37.5 to 1000 µg/mL. Cell viability was determined by the MTT assay and expressed as a percentage relative to the untreated negative control. Linear regression analysis was used to estimate IC_50_ values from the experimental dose–response data.

The biological performance of the scaffolds was evaluated using HEp-2 tumor cells and BHK-21 fibroblasts. The results revealed marked differences among the formulations. F3 exhibited the strongest cytotoxic effect against HEp-2 cells, with an estimated IC_50_ of 373.6 µg/mL, whereas F1 and F2 did not reach 50% inhibition within the tested concentration range (Figure 6). These findings suggest that the higher CUR and NPs-SiO_2_ content in F3 enhanced the biological response against tumor cells [66,67,68].

In contrast, F3 showed the most favorable response in BHK-21 fibroblasts, maintaining cell viability above 80% under the evaluated conditions. F1 and F2 produced greater reductions in fibroblast viability, particularly at higher concentrations. Therefore, among the evaluated formulations, F3 presented the most suitable balance between cytotoxicity toward tumor cells and compatibility with healthy fibroblasts [69,70]. The distinct cellular behavior observed for F3 may be associated with the combined contribution of CUR and silica nanoparticles within the hybrid matrix. However, additional mechanistic studies are necessary to identify the molecular pathways involved in these biological responses [71].

**Figure 7:**
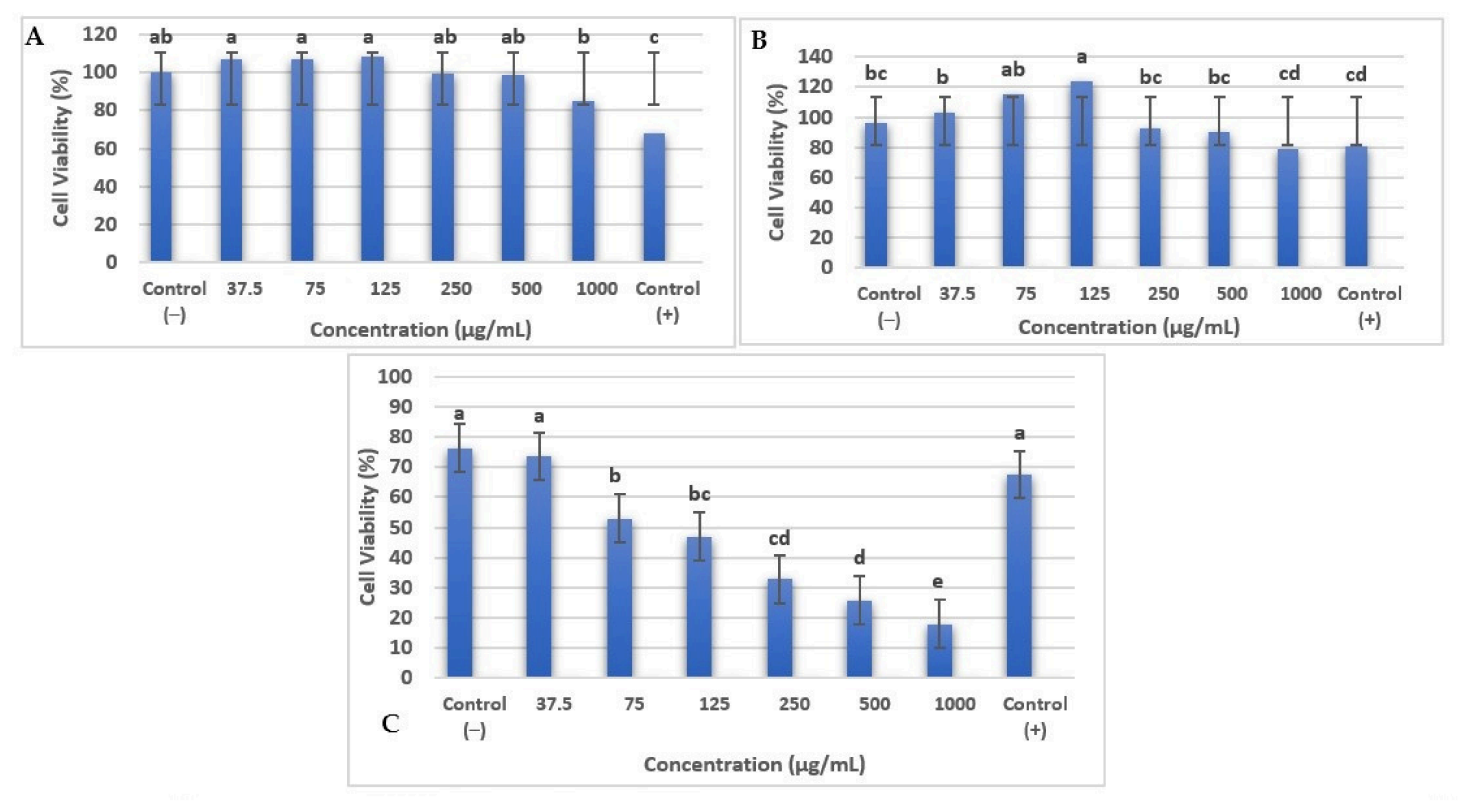
Effect of CUR/SiO_2_-doped formulations on HEp-2 cell viability. (**A**) F1, (**B**) F2, and (**C**) F3 treatments showing dose-dependent responses across concentrations (37.5–1000 µg/mL). Viability was normalized to the untreated control. Values represent mean ± standard deviation (SD). Statistical analysis was performed using one-way ANOVA followed by Tukey’s multiple comparison test. Distinct letters denote significant differences among groups (*p* < 0.05).

Figure 7 provides a detailed comparison of cell viability among treatments. F1 and F2 maintained viability values close to or above those of negative control at low and intermediate concentrations, whereas F3 produced a clear concentration-dependent reduction in viability. The strongest inhibitory effect was observed at 500 and 1000 µg/mL, with statistically significant differences relative to lower concentrations and the untreated control (*p* < 0.05). These findings confirm the greater cytotoxic activity of F3 toward HEp-2 cells.

Figure 7B shows the dose–response behavior of formulation F2 in the HEp-2 cell line, revealing a cytotoxic profile distinct from those observed for F1 and F3. These differences suggest that the simultaneous incorporation of CUR (1%) and NPs-SiO_2_ (1%) modifies the interaction between the cells and the CS/PVP/PVA polymer matrix. At low and intermediate concentrations (75–125 µg/mL), cell viability remained comparable to or slightly higher than that of the negative control, suggesting a possible biostimulatory effect associated with subcytotoxic concentrations of CUR and NPs-SiO_2_. At higher concentrations (250–1000 µg/mL), viability decreased moderately; however, it remained relatively high across the evaluated range, indicating limited cytotoxic activity against HEp-2 cells under the tested conditions. Consequently, F2 did not reach 50% inhibition within the evaluated concentration range, and the estimated IC_50_ value was extrapolated beyond 1000 µg/mL. Figure 7C shows the effect of F2 on HEp-2 cell viability. The highest percentages were observed at 75 and 125 µg/mL, whereas higher concentrations resulted in a gradual decrease in viability. However, statistically overlapping groups were identified among several treatments, suggesting partial similarity between concentrations. As illustrated in Figure 7C, F3 produced the strongest inhibitory effect on HEp-2 cell viability among the formulations evaluated.

**Figure 8:**
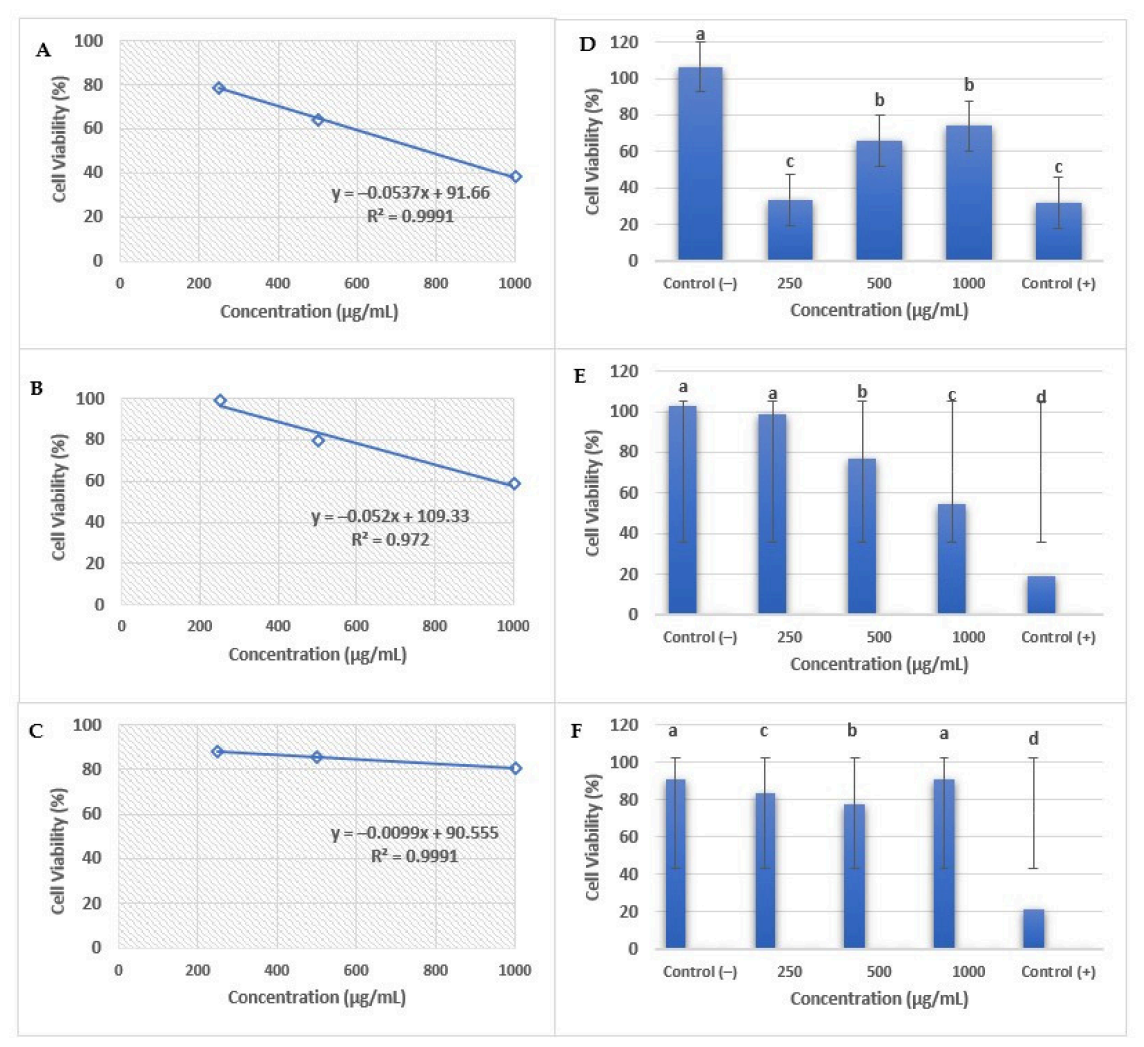
Cytotoxicity and cell viability evaluation of F1, F2, and F3 formulations in the BHK-21 cell line. (**A**–**C**) Dose–response curves showing the cytotoxic effects of F1 (**A**), F2 (**B**), and F3 (**C**) formulations, derived from linear regression analysis correlating treatment concentration (µg/mL) with cell viability (%). Regression equations and coefficients of determination (R^2^) are presented for each treatment. (**D**–**F**) Comparative analysis of cell viability following exposure to 250, 500, and 1000 ppm of F1 (**D**), F2 (**E**), and F3 (**F**), including negative and positive controls. Data are expressed as mean ± standard deviation (SD). Different letters above the bars indicate statistically significant differences among concentration groups within each formulation (*p* < 0.05), according to Tukey’s post hoc test.

Figure 8 summarizes the biological response of BHK-21 fibroblasts. F1 showed the greatest cytotoxicity, with an estimated IC_50_ of approximately 377 µg/mL and a marked reduction in viability at concentrations ≥250 µg/mL. F2 displayed intermediate behavior, whereas F3 maintained cell viability above 70–80% throughout the evaluated concentration range, consistent with the cytocompatibility criteria established in ISO 10993-5 [72]. These results indicate that F3 exhibited the most favorable biological response toward healthy fibroblasts.

### 2.9. In Vivo Preliminary Biocompatibility Study of Scaffolds Through Subdermal Implantations of the Different Formulations

The subcutaneous pocket without an implant, used as a reference for the normal healing process, showed complete wound closure at 30 days and no evident macroscopic alterations compared to the adjacent non-operated areas. Since no findings of interest for the study objective were observed, this group was not included in the histological figures presented.

#### 2.9.1. F1 Implantation

**Figure 9:**
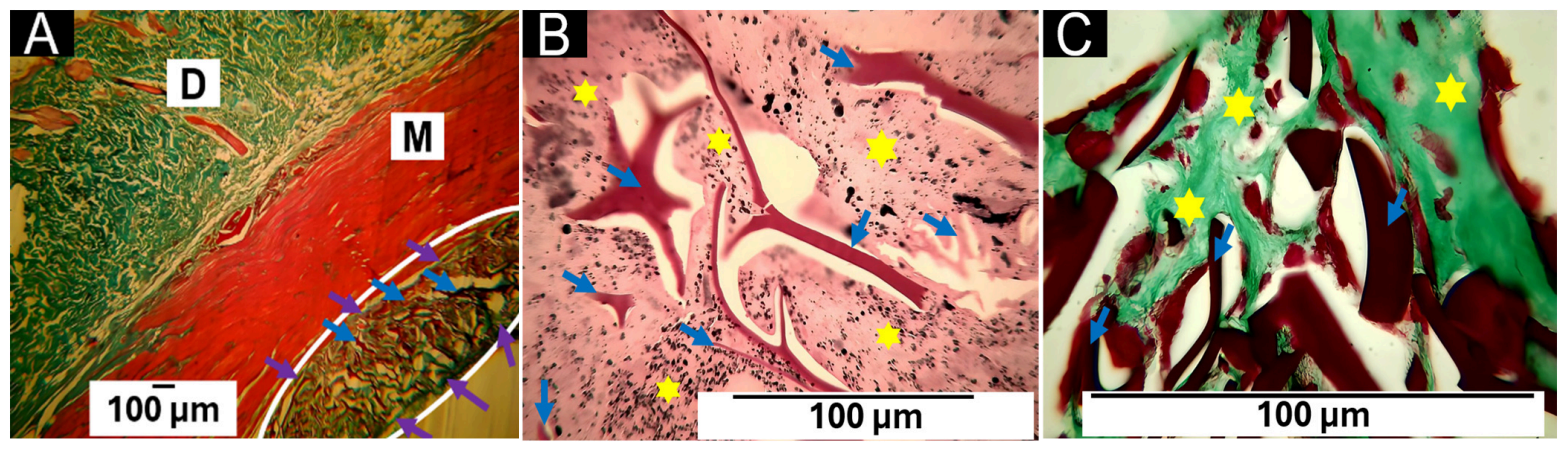
F1 system implanted subdermally for 30 days. (**A**) 4× image, GT technique. (**B**) 40× image, HE technique. (**C**) 100× image, GT technique. D: Dermis. M: Muscle. White oval: implantation site. Purple arrows: reticular fibers. Blue arrows: material fragments. Yellow star: collagen matrix.

Figure 9 presents representative histological images of the F1 system (40% PVP, 40% CS, and 20% PVA). In micrograph 9A (4×), the implantation area is clearly identified, delimited by a white oval. In this region, multiple dispersed fragments consistent with remnants of the implanted biomaterial are observed (blue arrows), embedded in loose connective tissue rich in reticular fibers. Gomori’s trichrome stain highlights these fibers and visualizes the organization of the extracellular matrix surrounding the material remnants [73].

A thin layer of reticular fibers (purple arrows) is observed around the implantation site, consistent with an incipient capsule that delimits the tissue–biomaterial interface. Its presence suggests an organized tissue response and an early process of connective tissue remodeling around the implant [74]. Figure 9B (40×) shows multiple residual fragments of the biomaterial (blue arrows) with different sizes and morphologies, consistent with progressive in situ degradation [75]. These remnants are surrounded by a collagen-rich matrix (yellow stars), which occupies the interstitial spaces and demonstrates the infiltration and organization of connective tissue around the material [76]. Figure 9C (100×) provides a more detailed view of the interaction between the biomaterial remnants and the surrounding tissue. The residual fragments exhibit varying degrees of fragmentation and are surrounded by a collagen-rich extracellular matrix. Connective tissue is also observed infiltrating the spaces previously occupied by the material, suggesting a progressive scaffold degradation process accompanied by tissue integration [76].

In subdermal rat biomodels, biomaterial implantation typically triggers a well-characterized physiological sequence that begins with an acute inflammatory response, followed by a proliferative phase dominated by fibroblastic activity and extracellular matrix deposition, and culminates in tissue remodeling [77]. In formulation F1, this progression was observed in an orderly manner, consistent with expectations for a biocompatible material [77]. The early formation of a collagen matrix around the implant fragments, along with a very thin fibrous capsule, indicates that the host tissue responded in a balanced manner, without triggering excessive inflammation or dense, restrictive encapsulation.

The observed histological pattern suggests that the material induced a controlled tissue response, allowing for the surrounding connective tissue to progressively occupy the spaces generated during scaffold degradation, without histological evidence of an exacerbated inflammatory response [78]. Thirty days after subdermal implantation, the F1 formulation (40% CS, 20% PVA, and 40% PVP) exhibited favorable integration and tissue response. A process of progressive scaffold resorption was observed, accompanied by the deposition of reticular collagen, which simultaneously served as transient support for inflammatory cells and as a natural scaffold for tissue reorganization. The extremely thin fibrous capsule surrounding the implantation site is a histological indicator of moderate, self-regulated inflammation, characteristic of materials that interact harmoniously with the biological environment [79]. Taken together, these findings indicate that F1 not only avoids chronic responses or marked encapsulation but also promotes an environment conducive to tissue repair, integration, and remodeling, solidifying its profile as a material with good biological acceptance.

#### 2.9.2. F2 Implantation

**Figure 10:**
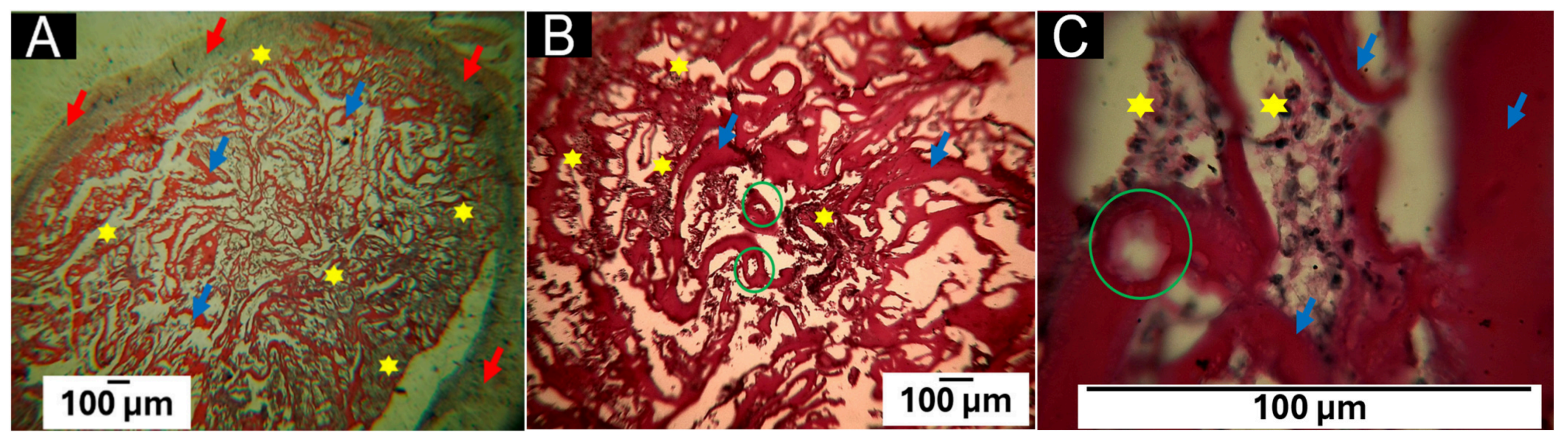
F2 system implanted subdermally for 30 days. (**A**) 4× magnification, GT technique. (**B**) 10× magnification, HE technique. (**C**) 100× magnification, HE technique. Red arrows: Fibrous capsule. Green circles: Fragments of the resorbed material. Yellow stars: Collagen matrix. Blue arrows: Fragments of the material.

Figure 10A shows a cross-section of the area where the F2 formulation was implanted, observed at 4× magnification. In this panoramic view, the complete outline of the implantation site is clearly visible, delimited by a thin fibrous capsule indicated by red arrows. This capsule, composed primarily of collagen fibers and spindle cells characteristic of remodeling connective tissue, represents an organized inflammatory response that acts as a regulated biological boundary between the implant and the host tissue [80]. Its uniform and minimally thickened morphology suggests that the body recognizes the material without generating excessive encapsulation, but rather as part of a physiological isolation mechanism that promotes controlled integration [81].

Within the implantation area, numerous intensely red-stained fragments are observed (blue arrows), corresponding to remnants of the biomaterial undergoing degradation. Their variability in size and distribution indicates a heterogeneous pattern of disintegration, consistent with the multicomponent nature of F2 and the simultaneous action of physicochemical and cellular processes. Among these fragments, several clear or slightly grayish areas, marked with yellow stars, also stand out. These are compatible with zones in which the material has been partially or completely degraded, thereby allowing cell influx and early extracellular matrix deposition. These transition areas (the boundary between residual biomaterial, cellular infiltration, and collagen synthesis) reflect an active biological process in which the host tissue begins to occupy the spaces left by implant degradation, thereby consolidating an environment conducive to tissue repair and the progressive integration of the scaffold [82].

Figure 10B, observed at 10× magnification, provides a clearer view of the internal architecture of the area implanted with formulation F2. At this level of detail, fragments of the biomaterial at various stages of fragmentation are identified and indicated by green circles. Their irregular morphology suggests an active degradation process mediated by both physicochemical phenomena and local cellular involvement. Surrounding these fragments is a more compact collagen matrix, indicated by yellow stars, which is progressively deposited and organized as the material loses structural continuity. This pattern, characterized by the coexistence of implant remnants and consolidating collagen, reflects a biological transition in which the host tissue begins to occupy the vacant spaces and gradually replace the degraded biomaterial. Figure 10C, obtained at the highest magnification (100×), clearly reveals the intimate interaction between the residual material and the surrounding tissue. In this image, fragments undergoing advanced resorption are visible, again indicated by a green circle. Their edges appear more irregular and eroded, consistent with active cellular degradation. A dense collagen matrix, marked with yellow stars, surrounds these fragments, and inflammatory cells and fibroblasts are integrated within it. The simultaneous presence of maturing collagen, active cells, and resorbing fragments suggests a healthy integration process, in which implant degradation occurs in a balanced manner alongside host tissue regeneration [61].

#### 2.9.3. F3 Implantation

**Figure 11:**
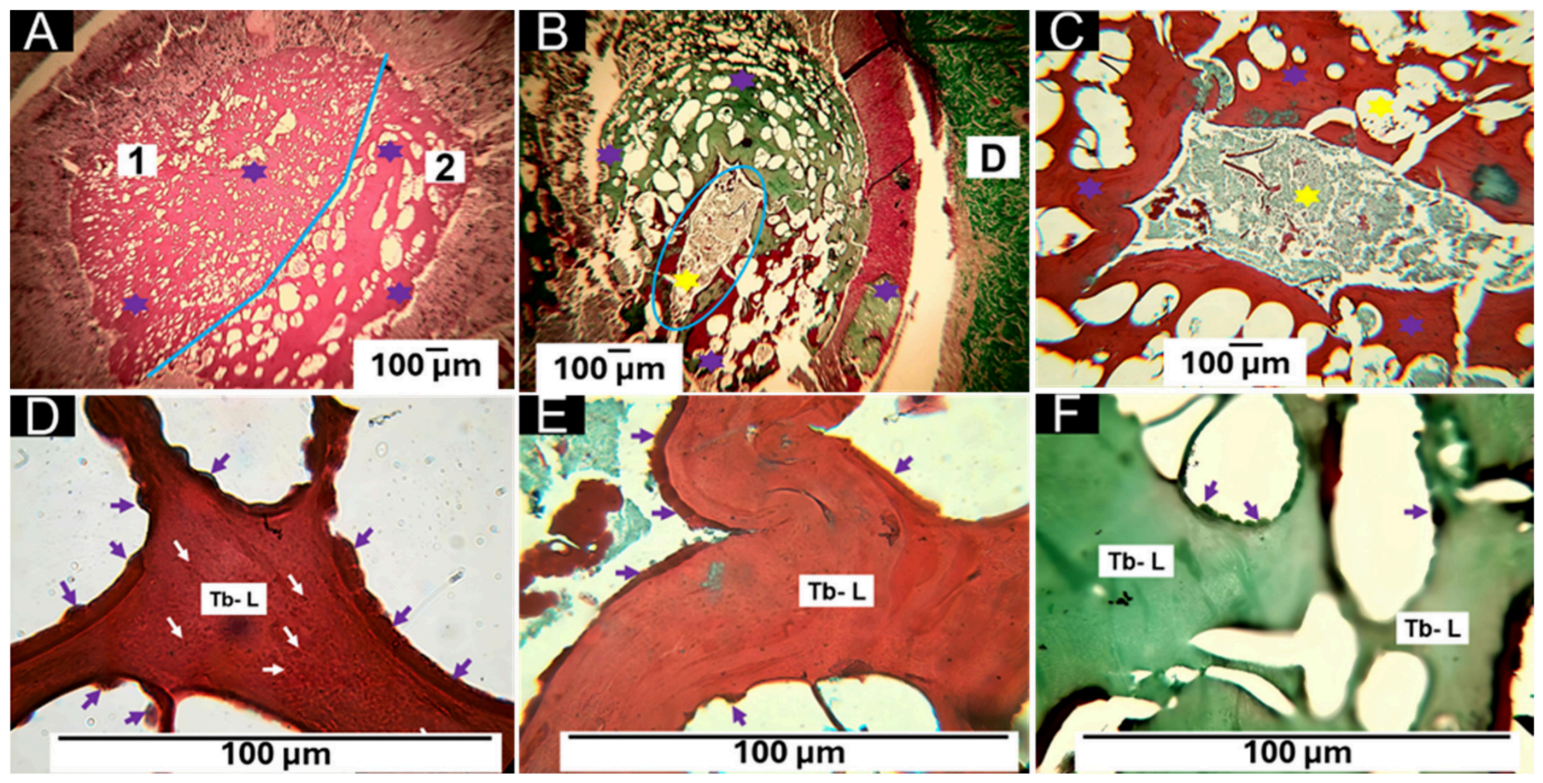
F3 system implanted subdermally for 30 days. (**A**) 4× image, HE technique. (**B**) 4× image, GT technique. (**C**) 10× image, GT technique. (**D**) 100× image, GT technique. (**E**) 100× image, GT technique. (**F**) 100× image, GT technique. Purple stars: Trabeculae-like bone structures. Numbers 1 and 2: Sample sectors. Blue line: Indicates the separation between sectors. Blue oval: Sector of histological interest. Yellow stars: Collagen matrix. Tb-L: Trabeculae-like bone structure. Purple arrows: structures with morphologies like those of osteoblasts.

The histological appearance of formulation F3 is clearly distinct from that observed in the other two formulations, indicating more complex tissue organization and an unusual remodeling pattern in subdermal models. In Figure 11A, a cross-section of the implantation site shows that the space originally occupied by the biomaterial has been replaced by reticular tissue, externally delimited by connective tissue, with no evidence of fibrous capsule formation, indicating a minimal inflammatory response and a permissive environment for tissue reorganization [79]. Within this reticular network, two sectors can be distinguished, separated by a blue line. The area identified as sector 1 comprises a network of numerous small pores, within which reddish, compact tissue is distributed. In contrast, sector 2 has larger but less abundant pores, and the interposed tissue exhibits an even greater density, with a histological pattern characterized by thicker and more organized matrix structures, suggestive of early tissue organization of an osteogenic type.

Figure 11B, corresponding to an additional section of the same biomodel, confirms this tissue organization. Again, the implanted region is surrounded by connective tissue without a fibrous capsule, supporting the notion of favorable biological integration. The tissue within the implantation area exhibits a reticular arrangement, stained green by the Gomori trichrome technique, indicating that it is primarily composed of reticular fibers and is consistent with a matrix rich in type III collagen, a characteristic of tissues in transition toward more mature structures [61]. Within this network, areas resembling developing bone trabeculae are identified and marked with purple stars, suggesting a three-dimensional organization process like that observed in the initial stages of bone development. Particularly striking is the presence of a central zone, delimited in blue and marked with a yellow star, which does not stain positively for GT and exhibits more compact tissue characteristics typical of primary tissue undergoing differentiation or maturation [83]. This pattern suggests that the central area may correspond to a matrix transitioning toward a more organized state, potentially consistent with the early stages of consolidation and maturation, as observed in more structured bone tissue.

Figure 11C, corresponding to the same region shown in Figure 11B but at 10× magnification, provides a clearer view of the tissue organization induced by the F3 formulation. In this image, a large central cavity is visible, filled with tissue that stains positively for reticular fibers, as evidenced by the characteristic green Gomori staining. This central tissue suggests the presence of a matrix rich in type III collagen, characteristic of early organizational processes or microenvironments transitioning toward more complex structures [83]. Surrounding this cavity are several red-stained areas, within which smaller, irregular cavities are observed. These cavities show cells distributed both peripherally and internally, and some contain green-stained reticular tissue, indicating active remodeling and differentiation [77].

The behavior is even more evident in Figure 11D–F, which are taken at 100× magnification. These images allow for detailed observation of several structures that, at lower magnification, were suggested to be possible early bone trabeculae. Within them, small cavities indicated by white arrows are identified, consistent with osteocytic lacunae, whereas on the surface of these structures (TB-L), rounded or slightly cuboidal cells, marked with purple arrows, are distinguished, resembling osteoblast-like morphology [79]. The combination of internal cavities, a dense matrix, and cells organized at the periphery reinforces the presence of morphological characteristics suggestive of early osteogenic tissue organization [84]. F1, composed of CS, PVA, and PVP, showed a progressive degradation process accompanied by the formation of an organized collagen matrix, along with a very thin and uniform fibrous capsule [85]. This pattern indicates a moderate inflammatory response and stable tissue integration, without signs of osteogenic differentiation, consistent with biofunctional polymer-based materials that lack specific inducers. F2, which incorporates small quantities of curcumin and silica nanoparticles, showed intermediate behavior. The collagen matrix was more abundant, and the degree of cellular integration was greater, suggesting that these additives modulated the inflammatory response and promoted matrix deposition, although they did not provide sufficient conditions to induce bone formation. The more pronounced fibrous capsule in F2 can be interpreted as a mechanism by which host tissue delimits the implant, a typical sign of a moderate response to foreign bodies [86].

The results for F3 were particularly striking. This system, with higher concentrations of CUR and NPs-SiO_2_, generated structures such as bone trabeculae, small cavities morphologically resembling osteocytic lacunae, and cells exhibiting osteoblastoid morphology, as well as cells compatible with osteoblasts on its surface, a rare phenomenon in subdermal implants. Since these structures were observed in only one biomodel, the findings should be considered preliminary and interpreted with caution. This finding suggests that the combination of both additives at higher levels may have contributed to the emergence of tissue structures morphologically consistent with early osteogenic tissue organization. However, these observations are based solely on histological morphology [86]. Previous studies have shown that CUR and silica-based materials can influence processes associated with osteogenic differentiation and the formation of mineralized tissue [87]. Although specific osteogenic markers were not evaluated in this study, and molecular characterization was not performed, the unusual histological organization observed in formulation F3, absent in the other formulations, warrants further investigation to elucidate the biological mechanisms involved.

These molecules appear to have generated a bioactive synergy potentially associated with osteogenic-like tissue organization. The presence of abundant reticular fibers, evidenced by Gomori trichrome staining in the implanted area, along with the trabecular arrangement of the observed structures, constitutes morphological finding consistent with early osteogenic tissue organization in formulation F3 [88]. Although ectopic bone formation in a subdermal environment is uncommon due to the scarcity of osteoprogenitor cells and limited vascularization, this case suggests that the composition and proportions of bioactive components could generate a microenvironment that favors the development of tissue structures not observed in other formulations.

## 3. Materials and Methods

### 3.1. Synthesis of Silicon Dioxide Nanoparticles (NPs-SiO_2_)

The NPs-SiO_2_ used in this study were synthesized according to the methodology previously described by Niebles Navas et al., which involves a sol–gel process in which tetraethyl orthosilicate (TEOS) is hydrolyzed and condensed in an alkaline medium containing hexadecyltrimethylammonium bromide (CTAB) and NH_4_OH. The resulting nanoparticles were purified by centrifugation, washed with distilled water and ethanol, and finally calcined at 250 °C. Preliminary physicochemical characterization showed that the NPs-SiO_2_ had a predominantly spherical morphology with an average particle size of 12.19 ± 0.13 nm [35].

### 3.2. Evaluation of the Minimum Inhibitory Concentration (MIC) of Nanoparticles

Six successive dilutions of the nanoparticles were prepared (3.0 mL of the previous stock and 3.0 mL of distilled water) as shown in Table 4. Four bacterial strains (*E. hormaechei*, *K. variicola*, *S. enterica*, and *S. aureus*) were selected and grown in nutrient broth for 24 h. The cells were centrifuged at 6000 rpm for 5 min, then washed with sterile 0.85% saline solution by vortexing for 1 min. This washing process was repeated three times. Finally, the cells were resuspended in saline to a final concentration of 10^8^ CFU/mL, as determined by optical density (OD_600_) using the previously established McFarland scale (0.5 McFarland standard).

In a 96-well polystyrene BRAND plate, 100 µL of the bacterial cell suspension and 100 µL of the NP solution were inoculated (Figures S1 and S2). The plates were incubated at 37 °C for 20 h. At the end of the incubation period, 100 µL of nutrient broth supplemented with 0.2% triphenyltetrazolium chloride (TTC) was added to each well, and the plates were incubated at 37 °C for 4 h. The minimum inhibitory concentration (MIC) was determined as the lowest concentration at which no red coloration was observed due to the indicator (as shown in Table 4). To determine the minimum bactericidal concentration (MBC) of the nanoparticles, 100 µL was taken from the wells where no change was detected in the TTC indicator, plated onto Mueller–Hinton agar plates, and incubated at 37 °C for 48 h. The MIB was the concentration at which no bacteria were detected after incubation.

### 3.3. Inhibitory Effect of CUR and NPs-SiO_2_ on Biofilm Production

The biofilms formed in the wells were stained with a crystal violet solution (0.1%) for 30 min (Figures S3 and S4). The plate was then washed again with deionized water to remove excess crystal violet that did not adhere to the biofilm. The dye bound to the biofilm was dissolved by adding 200 µL of 30% acetic acid, and its optical density (OD_590_) was measured at 590 nm using a spectrophotometer (Biospectrometer, Eppendorf AG, Wandsbek, Germany). The percentage of biofilm inhibition was calculated relative to the biofilm produced by the bacterial strain in the absence of NPs.

### 3.4. Synthesis of PVA/PVP/CS/CUR/NPs-SiO_2_ Scaffolds

To prepare the hybrid scaffolds, we used polyvinyl alcohol (PVA), polyvinylpyrrolidone (PVP), chitosan (CS), curcumin (CUR), and silicon dioxide nanoparticles (NPs-SiO_2_). The procedure followed an adaptation of previously established methodologies [35]. The PVA employed in the formulations was of a highly hydrolyzed grade (≥99% hydrolysis; molecular weight 85,000–124,000 g·mol^−1^) obtained from Sigma-Aldrich (St. Louis, MO, USA). Polyvinylpyrrolidone (PVP; Mw ≈ 40,000 g·mol^−1^) was also sourced from Sigma-Aldrich (St. Louis, MO, USA). Chitosan with a degree of deacetylation above 80% and viscosity between 20 and 300 cP (corresponding to a low-molecular-weight grade) was purchased from Sigma-Aldrich (St. Louis, MO, USA). CUR (≥94%, Sigma-Aldrich, St. Louis, MO, USA) was incorporated as the bioactive component and obtained from the same supplier.

First, 8 g of chitosan was dissolved in 200 mL of 2% (*v*/*v*) acetic acid under constant stirring. Simultaneously, aqueous solutions of 8 g PVP in 200 mL, 2.40 g PVA in 60 mL, and 0.20 g CUR in 5 mL of ethanol were prepared, all under continuous stirring. Additionally, 0.15 g of NPs-SiO_2_ was dispersed in 5 mL of distilled water using an ultrasonic bath (Branson, Madrid, Spain) for 90 min (Table 5). Subsequently, all solutions were mixed and sonicated to ensure system homogeneity, as detailed in Table 5. The three formulations were lyophilized using a Labconco FreeZone^®^ (Labconco Corporation, Kansas City, MO, USA) model 6 Liter Benchtop unit, operating at 0.05 mbar and −50 °C for 72 h to remove residual solvents and obtain the xerogel while preserving the molecular integrity of the crosslinked material.

#### 3.4.1. Fourier Transform Infrared Spectroscopy (FTIR)

The chemical nature of the materials was analyzed using Fourier transform infrared spectroscopy (PerkinElmer Inc., Waltham, MA, USA) with attenuated total reflection (ATR-FTIR) on a PerkinElmer Spectrum Two instrument (PerkinElmer Inc., Waltham, MA, USA). FTIR spectra were taken in the range of 4000–450 cm^−1^ with a resolution of 4 cm^−1^, accumulation of 16 scans, and a step increment of 1 cm^−1^ [89].

#### 3.4.2. X-Ray Diffraction (XRD)

The crystalline structure of the scaffolds was characterized by X-ray diffraction (XRD) using a Bruker D8 ADVANCE diffractometer equipped (Bruker Corporation, Billerica, MA, USA) with Cu Kα radiation (λ = 1.5406 Å) and operated over a 2θ range of 5–80°.

#### 3.4.3. Electron Microscopy (SEM)

The morphology of the lyophilized scaffolds was analyzed by scanning electron microscopy (SEM) using a Zeiss EVO10 microscope (Carl Zeiss Microscopy GmbH, Jena, Germany). Before SEM analysis, the samples were coated with gold using the Cressington 108 Auto Sputter Coater (Carl Zeiss Microscopy GmbH, Jena, Germany) operated at 22 mA for 1 min. The scaffolds were coated with gold to enhance electronic conductivity, and the SEM images revealed a porous structure with homogeneously distributed NPs-SiO_2_.

Additionally, quantitative porosity analysis was performed on 500× micrographs, with at least 70 pores measured, and image processing was carried out in ImageJ (v1.54, National Institutes of Health, Bethesda, MD, USA) to obtain numerical data [90]. Since the materials exhibited irregular porosity, the diameter was calculated from the Feret diameter (µm) and the Feret/MinFeret ratio, which is used as an elongation descriptor (these geometric parameters are commonly used to describe the size of irregular objects), a practice very common in studies of this type [91]. Because the data did not follow a normal distribution, a statistic for a skewed distribution, such as the Shapiro–Wilk test, was used, and homogeneity of variances was verified with Levene’s test (*p* > 0.05). Global comparisons between formulations (F1, F2, and F3) were performed using the non-parametric Kruskal–Wallis test [92], followed by a Dunn post hoc analysis with Bonferroni correction for pairwise comparisons [93].

In addition, energy-dispersive X-ray spectroscopy (EDS) was performed to determine the elemental composition and spatial distribution of the three formulations (F1, F2, and F3). Before analysis, the formulations were fixed to aluminum supports using conductive carbon tape and coated with a thin layer of gold by sputtering to improve surface conductivity and minimize charge effects under the electron beam [94]. Measurements were carried out using a scanning electron microscope (SEM) (EVO10, Carl Zeiss Microscopy GmbH, Jena, Germany) equipped with an EDS detector, operating at a typical accelerating voltage of 20 kV, suitable for polymeric biomaterials. Spectra were acquired from representative surface regions of the formulations, including both pore walls and internal structures, to ensure the statistical reliability of the compositional analysis. In this regard, qualitative and semi-quantitative elemental analyses were performed to identify key elements, such as carbon (C), oxygen (O), and nitrogen (N), characteristic of the formulations and their associated polymeric components [95]. Elemental mapping was also conducted to evaluate the spatial distribution of these elements within the formulation matrix. EDS data were processed using specialized analytical software, applying ZAF or Phi-Rho-Z corrections to improve quantitative accuracy. Due to the inherent limitations of EDS for light elements and organic matrices, the results were primarily interpreted comparatively and qualitatively, focusing on the relative elemental distribution rather than absolute concentration values [96].

Therefore, SEM-EDS results were used to assess the relative elemental distribution of the scaffold components within the analyzed regions. However, this technique was not intended to quantify the actual retention efficiency of CUR or NPs-SiO_2_ after scaffold processing, and the reported compositions should be interpreted as semiquantitative rather than absolute measurements.

#### 3.4.4. Analysis of the Mechanical Properties of PVA/CS/NPs-SiO_2_ Scaffolds

The mechanical properties were evaluated by compression testing of the scaffolding using a SHIMADZU EZ-LZ universal testing machine (Shimadzu, Tokyo, Japan). This machine was operated with a 500 N load cell. Cylindrical scaffolds 20 mm wide were analyzed in triplicate. A compression speed of 10 mm/min was used.

#### 3.4.5. Thermal Analysis of CS/PVP/PVA/CUR/NPs-SiO_2_ Scaffolds

Differential scanning calorimetry (DSC) measurements were carried out using a Mettler Toledo DSC1/500 instrument (Mettler-Toledo AG, Greifensee, Switzerland). Samples were heated from −25 °C to 200 °C in a nitrogen atmosphere at 10 °C/min, then held at 250 °C for 10 min to stabilize, and finally cooled to 0 °C. Then, a second heating cycle was performed from 0 °C to 250 °C. Differential scanning calorimetry (DSC) analysis was performed starting with the second heating cycle to eliminate interference from residual water and the thermal history induced by freeze-drying, ensuring that the observed transitions correspond to the intrinsic structure of the material rather than moisture. Thermogravimetric analyses (TGAs) were conducted using a TGA 2 (Mettler Toledo, Singapore) setup under a nitrogen-inert atmosphere (50 mL/min). Samples were placed in alumina crucibles and heated from 25 °C to 900 °C at a heating rate of 10 °C/min [97].

### 3.5. Evaluation of the Antimicrobial Activity of the Scaffolds

Four bacterial strains (*E. hormaechei*, *K. variicola*, *S. enterica*, and *S. aureus*) were selected and grown in nutrient broth for 24 h. Then, the cells were centrifuged at 6000 rpm for 5 min and washed with sterile 0.85% saline solution by vortexing for 1 min. This washing process was repeated three times. Finally, the cells were dispersed in saline solution to reach a final concentration of 10^6^ CFU/mL using the optical density (OD_600_) and the previously established McFarland scale (0.5 McFarland standard).

One hundred microliters of the strain were impregnated onto a 0.25 cm^3^ (1.0 × 0.5 × 0.5) piece of scaffolding and placed in a test tube, which was incubated for 24 h at 37 °C. A control was prepared by replacing the scaffolding with cellulose filter paper (Whatman No. 1). At the end of the incubation period, 1.0 mL of 0.85% saline solution was added, the mixture was allowed to stand for 1 h and was then vortexed for 1 min to detach the cells from the scaffold. Six successive dilutions were then prepared in nutrient broth supplemented with 0.2% triphenyltetrazolium chloride (TTC). Then, 200 µL of each dilution was placed in the wells of 96-well polystyrene microplates (BRANDplates) and incubated at 37 °C for 24 h. At the end of the incubation period, a color change in the indicator was recorded as a positive test. Where no color change was detected in the TTC indicator, the cells were plated onto Mueller–Hinton agar plates and incubated at 37 °C for 48 h to verify inhibition of the microorganism. Four replicates were performed for the analysis.

### 3.6. Evaluation of Cytotoxicity and Cell Viability

HEp-2 (ATCC CCL-23) and BHK-21 [C-13] (ATCC CCL-10) cell lines were provided by the Laboratorio de Investigaciones de Química y Biología, Universidad del Norte, Barranquilla, Colombia. Cells were maintained under standard culture conditions and used for the cytotoxicity and cell viability assays described below. For cell viability assessment, scaffolds were prepared at a concentration of 1000 ppm per sample and placed in Falcon tubes, sterilized by exposure to ultraviolet light in a laminar flow cabinet for biological products for 24 h (Purifier™ Cell Logic™+, Madrid, Spain). Each scaffold was immersed in 10 mL of Roswell Park Memorial Institute (RPMI) medium supplemented with 2% penicillin–streptomycin (P/S) and 10% fetal bovine serum (FBS). Samples were incubated on an OrbiCult™ IBS orbital shaker (IBS-NR, Singapore) at 37 °C and 200 rpm.

For the MTT assay (3-(4,5-dimethyl-2-thiazolyl)-2,5-diphenyl-2H-tetrazolium bromide), 100 µL of cell suspension from the HEp-2 and BHK-21 cell lines was seeded into 96-well plates at an initial density of 10,000 cells per well. Four wells were used per sample, with serial dilutions and replicates for each formulation, and twelve wells were designated for the negative control (RPMI medium without scaffolding). The plates were incubated at 37 °C and 5% CO_2_ for 20 h to allow for cell adhesion. Subsequently, 0.5% MTT (Sigma-Aldrich, St. Louis, MO, USA) was added to the FBS, and the medium was incubated for 3 h under the same conditions. After removing the medium, 100 µL of dimethyl sulfoxide (DMSO) was added directly to each well to solubilize the formazan crystals. Blank wells containing all reagents (medium, MTT, and DMSO) but without cells were included to correct for baseline absorbance. The plates were shaken for 10 min, and the optical density was measured at 570 nm using a FLUOstar Omega microplate reader (BMG LABTECH, Ortenberg, Germany). Cell viability was calculated using the following equation, where DO stands for Optical Density (absorbance).

(1) $$Viability\;\left(\%\right)=\frac{{DO}_{sample}-{DO}_{Blank}}{{DO\;}_{Control}-{DO}_{Blank}}$$

### 3.7. In Vivo Biocompatibility Assessment

Three four-month-old male Wistar rats (average weight: 280 g) from the LABBIO animal facility at Universidad del Valle were used. The sample size was determined in accordance with ISO 10993-6 [98] and the reduction principle. All procedures were approved by the Animal Experimentation Ethics Committee of Universidad del Valle (CEAS), Resolution No. 006-022, dated 5 September 2022, and were performed in accordance with ARRIVE 2.0 guidelines. In contrast, the fourth pocket remained unplanted as a reference for normal healing. The animals were anesthetized using a ketamine and xylazine protocol. After preparation of the dorsal region, four independent subcutaneous pockets were created. Formulations F1, F2, and F3 were implanted into three of the pockets, while the fourth pocket remained unplanted as a reference for normal healing. The wounds were closed with 4-0 Vicryl^®^ sutures, and the animals received postoperative analgesia and daily clinical monitoring. After 30 days, the biomodels were euthanized by sodium pentobarbital overdose. The implants and surrounding tissues were retrieved, fixed in 10% buffered formalin, and processed for histological analysis using hematoxylin and eosin (H&E) and Gomori trichrome (GT) staining. The evaluation included the inflammatory response, tissue organization, extracellular matrix deposition, material degradation, and tissue–biomaterial interaction. The details of the surgical procedure and histological processing have been described in previous research [73,99]. No postoperative complications or mortality were recorded during the experimental period.

Three adult male Wistar rats were used for the in vivo evaluation. Each biomodel received a sample of each experimental formulation (F1, F2, and F3), implanted into anatomically equivalent dorsal subcutaneous pockets, along with an unplanted pocket as a negative control. Formal randomization was not performed because all formulations were implanted into each biomodel using the same anatomical scheme to standardize the experimental procedure and reduce interindividual biological variability. Blinding was not applied during the surgical procedures or during the implantation of the biomaterials; however, the histological evaluation was performed without knowledge of the specific chemical composition of each formulation. The in vivo study was exploratory and descriptive; therefore, the histological findings were evaluated qualitatively, and inferential statistical analyses were not performed due to the small number of biomodels. Consequently, the multiple implantation sites within the same biomodel were not treated as independent statistical samples; rather, the observations were interpreted descriptively by comparing the tissue response associated with each formulation across the three biomodels.

### 3.8. Statistical Analysis

Statistical analysis of the results from this research (including mechanical resistance and antimicrobial activity tests) was conducted to ensure the comparative validity of the formulations studied. The statistical analysis of porosity obtained by SEM from the three scaffolding formulations was performed considering the data distribution and the assumptions of normality and homogeneity of variances. Normality was assessed using the Shapiro–Wilk test, and homogeneity of variances was verified with Levene’s test (*p* > 0.05). The data exhibited positive skewness, thus precluding the use of parametric tests. Consequently, overall comparisons among the formulations (F1, F2, and F3) were performed using the non-parametric Kruskal–Wallis test, followed by a Dunn post hoc analysis with Bonferroni correction for pairwise comparisons.

On the other hand, for mechanical tests, the means obtained in each test were subjected to an analysis of variance (ANOVA), which identified overall differences between treatments. Once significant differences were detected, the data were grouped using Fisher’s least significant difference (LSD) method. In addition, a linear regression analysis was conducted to assess the internal consistency of the results and to determine the degree of fit among the mechanical variables evaluated across the three treatments of CS/PVP/PVA/CUR/NPs–SiO_2_ scaffolds. This approach enabled estimation of the model’s predictive power and verification of the reliability of the mechanical response patterns under changes in scaffold composition.

Similarly, cell viability was expressed as a ratio relative to negative control, set as 100% baseline viability. This allowed for homogenization of responses across plates and eliminated variations inherent to the experimental system, facilitating direct comparison between treatments. From these values, the percentages of cell inhibition were calculated as a complementary variable. Before inferential analysis, the normality of the data distribution was assessed using normality tests such as the Shapiro–Wilk test. Subsequently, a one-way analysis of variance (ANOVA) was conducted for each formulation, with composition as the independent variable and cell viability percentage as the dependent variable. This procedure allowed for us to study whether there were statistically significant differences between the experimental groups. The validity of the model was supported by comparing the calculated F-statistic with the critical value and the associated significance level (*p* < 0.05), the criterion under which the null hypothesis of equal means was rejected.

To specifically identify which groups differed from one another, a post hoc multiple comparisons test was conducted using Tukey’s HSD method. This analysis was based on the mean squared error (MSE) from ANOVA and the Studentized Range distribution, thereby establishing a critical threshold (HSD) for comparing absolute differences between means.

Additionally, the dose–response relationship was analyzed using mathematical models. Where the data distribution allowed, the IC_50_ value was estimated by linear interpolation between the experimental points closest to 50% inhibition. This parameter was interpreted as a quantitative indicator of cytotoxic potency. All analyses were performed using Minitab software (2019) (v19.1, Minitab LLC, State College, PA, USA).

## 4. Conclusions

In this research, we synthesized three scaffold formulations based on CS, PVA, and PVP hydrogels, incorporating NPs-SiO_2_ as a functional inorganic phase. The system was then lyophilized to obtain the final xerogels. Structural characterization included Fourier transform infrared spectroscopy (FTIR), X-ray diffraction (XRD), and thermogravimetric analysis (TGA), approaches that allowed for us to identify how the incorporation of CUR and NPs modifies the material’s architecture without compromising its fundamental and functional integrity.

FTIR analysis confirmed the formation and stability of the polymer network across all formulations, regardless of compositional variations. The preservation of the main characteristic bands indicates that the incorporation of CUR, evidenced by the appearance of bands associated with vibrations of -OH groups (~3200–3500 cm^−1^), -COOH groups (~1600 cm^−1^ and ~1500 cm^−1^) and aromatic double bonds (~1420–1450 cm^−1^), and of NP-SiO_2_, evidenced by vibrations of Si–O–Si bonds (~1080–1100 cm^−1^) and Si–OH bonds (~3200–3600 cm^−1^), and that the doping or inorganic filling does not compromise the fundamental chemical structure of the matrix. The spectral changes observed in formulations F2 and F3 suggest molecular-level rearrangements associated with polymer–filler interactions, supporting the effective incorporation of both the CUR and the NP-SiO_2_. However, these variations remain within a range consistent with a structurally coherent system, reinforcing the conclusion that the hybrid formulations maintain their integrity even when incorporating functional additives.

The study of the mechanical properties of the three formulations revealed that F2 exhibited the highest values for stiffness and compressive strength. At the same time, F3 exhibited the most balanced behavior in flexibility, displacement, and structural stability (Table 1), which are essential attributes for scaffolds designed to support variable loads without compromising their integrity. Similarly, all formulations demonstrated complete antimicrobial activity against the evaluated strains, ensuring a bacterial-contamination-free environment during the critical phases of tissue integration. Similarly, biological analysis revealed statistically significant differences between formulations. Quantitative assessment of cell viability, based on normalized absorbance data, showed a clear concentration-dependent response, as further confirmed by a one-way ANOVA (*p* < 0.05). For F1, post hoc analysis (Tukey) demonstrated that the lowest concentration (250 ppm) exhibited cytotoxic effects comparable to the positive control. In contrast, intermediate and high concentrations showed partial recovery of viability, indicating a non-linear response pattern. In contrast, F2 induced a progressive reduction in cell viability as concentration increased, although the inhibitory effect did not reach 50% within the evaluated range, suggesting limited cytotoxic potency. Conversely, F3 maintained viability levels that were statistically indistinguishable from the negative control at low concentrations, while showing only moderate reductions at higher concentrations, supporting its biocompatibility. Estimated IC_50_ values reinforced these observations. Specifically, F1 exhibited intermediate cytotoxicity (~377 ppm), whereas F2 and F3 did not reach effective inhibitory concentrations within the tested range (IC_50_ > 1000 ppm), indicating a lesser impact on normal cell viability. These values support the observed cytocompatibility trends, which are critical for in vivo applications.

These observations suggest tissue responses associated with early osteogenic-type organization, highlighting the behavior of the F3 formulation. These results position F3 as a suitable bioactive platform that integrates structural stability, antimicrobial activity, biocompatibility with healthy cells, selectivity against tumor cells, and, notably, the ability to induce advanced tissue organization phenomena, including morphological features suggestive of early osteogenic-like tissue organization. These attributes make F3 a promising candidate for applications in tissue engineering and regeneration, particularly in scenarios where not only structural support is required but also active biological stimulation towards complex repair processes.

## Figures and Tables

**Table 1:** Morphometric analysis of pores in freeze-dried scaffolds, obtained by scanning electron microscopy (SEM).

Formulation	Diameter	Elongation
F1	* 12.68 [7.61–21.07] (23.34 ± 31.31)	* 1.917 [1.536–2.483] (2.336 ± 1.184)
F2	* 9.68 [7.32–18.07] (18.13 ± 22.97)	* 1.711 [1.402–2.163] (1.906 ± 0.783)
F3	* 17.81 [10.79–38.06] (29.99 ± 29.67)	* 1.891 [1.595–2.395] (2.084 ± 0.782)

Note *: Data are presented as median [Q1–Q3] (mean ± SD). Q1: 25th percentile; Q3: 75th percentile; SD: standard deviation.

**Table 2:** Mechanical properties of CS/PVP/PVA/CUR/NPs-SiO_2_ scaffolds. Mechanical properties data were reproduced from [26] licensed under CC BY 4.0.

Formulation	Elastic Modulus MPa *	Max. Strength (N) *	Max. Compressive Stress MPa *	Max. Displacement (mm) *	Max. Deformation (%) *
F1	0.236 ± 0.053 ^b^	332.786 ± 7.143 ^ba^	0.865 ± 0.114 ^a^	21.438 ± 0.763 ^a^	87.156 ± 3.634 ^a^
F2	0.773 ± 0.177 ^a^	398.968 ± 11.672 ^a^	1.015 ± 0.012 ^a^	23.649 ± 0.559 ^a^	86.373 ± 1.702 ^a^
F3	0.330 ± 0.115 ^b^	374.078 ± 46.188 ^ab^	0.848 ± 0.161 ^a^	23.159 ± 1.524 ^b^	87.849 ± 0.870 ^a^

* Different letters appearing as superscripts in the same column indicate significant differences (*p* < 0.005). Averages that do not share a letter are significantly different.

**Table 3:** Evaluation of the antimicrobial activity and Biofilm inhibition of NPs-SiO_2_ and CUR against Gram-positive and Gram-negative bacterial strains.

Strain	NPs-SiO_2_				CUR			
	MIC (mg/mL)	MBC (mg/mL)	Sub-MIC (mg/mL)	Biofilm Inhibition (%)	MIC (mg/mL)	MBC (mg/mL)	Sub-MIC (mg/mL)	Biofilm Inhibition (%)
*E. hormaechei*	>60	ND	60	58.6 ± 3.7	15.0 ± 0.1	15.0 ± 0.1	7.5	(−) 17.7 ± 1.1
*K. variicola*	>60	ND	60	56.1 ± 5.1	30.0 ± 0.2	ND	15.0	46.3 ± 4.2
*S. enterica*	>60	ND	60	42.5 ± 3.9	15.0 ± 0.1	30.0 ± 0.2	7.5	82.6 ± 7.5
*S. aureus*	>60	ND	60	72.5 ± 4.5	1.9 ± 0.1	7.50 ± 0.05	0.94	92.8 ± 5.7

**Table 4:** Evaluation of the minimum inhibitory concentration of NPs-SiO_2_ and CUR.

			Well Concentration (mg/mL)					
Sample	Weight (mg)	Volume (mL)	6	5	4	3	2	1
NPs-SiO_2_	180	3	60.00	30.00	15.00	7.50	3.75	1.88
CUR	180	6	30.00	15.00	7.50	3.75	1.88	0.94

**Table 5:** Solutions obtained by mixing specific proportions until a final concentration of 4% (*w*/*v*) is reached.

Components	F1 (%)	F2 (%)	F3 (%)
PVP	40	40	40
CS	40	40	40
PVA	20	18	16
CUR	-	1	2
NPs-SiO_2_	-	1	2

## Data Availability

The data supporting the findings of this study are available within the article and its Supplementary Materials. FTIR and TGA figures, mechanical properties, and antimicrobial scaffolds data were previously presented in preliminary form as part of a conference poster at the 5th International Online Conference on Nanomaterials (Session: Nanomedicine and Bionanotechnology) [26]. The present manuscript substantially expands upon these preliminary findings through additional characterization, biological evaluation, data analysis, and interpretation. All reused material is properly cited and complies with the terms of the original publication license. Reproduced from [26], licensed under CC BY 4.0.

## Supplementary Materials

The following supporting information can be downloaded at https://www.mdpi.com/article/10.3390/ijms27156856/s1.
