# Supplementary material for: Freeze-Dried CS/PVP/PVA Composite Scaffolds Doped with Curcumin and SiO2 Nanoparticles for Tissue Regeneration
Source: Int J Mol Sci. 2026 Jul 30;27(15):6856. doi: 10.3390/ijms27156856 (PMC13466538; doi:10.3390/ijms27156856)

International Journal of *Molecular Sciences*

MDPI

Supporting information of

Freeze-Dried CS/PVP/PVA Composite Scaffolds Doped with Curcumin and SiO_2 Nanoparticles for Tissue Regeneration

Domingo Cesar Carrascal-Hernández[1]*, Jairo Ortiz[2], Alexander Córdoba[3], Paula A. Zapata[3], Carlos Humberto Valencia-Llano[4], Diego López-Tenorio[4], Diana Paola Navia-Porras[5], Johannes Delgado-Ospina[5], Eliceo Cortes[1], Juan David Rodríguez Macías[6] and Carlos David Grande-Tovar[2]*.

[1] Grupo de Investigación en Sociedad, Educación y Desarrollo Humano (GISEDH). Facultad de Ciencias, Educación, Artes y Humanidades. Institución Universitaria de Barranquilla, Barranquilla, 080002, Colombia

[2] Grupo de Investigación de Fotoquímica y Fotobiología, Programa de Química, Universidad del Atlántico, Carrera 30 No. 8–49, Puerto Colombia, 081007, Colombia

[3] Grupo Polímeros, Departamento de Ciencias del Ambiente, Facultad de Química y Biología, Universidad de Santiago de Chile (USACH), Santiago 9170022, Chile

[4] Grupo Biomateriales Dentales, Escuela de Odontología, Universidad del Valle, Calle 4B # 36-00, Cali, 76001, Colombia

[5] Grupo de Investigación Biotecnología, Facultad de Ingeniería, Universidad de San Buenaventura Cali, Carrera 122 # 6-65, 76001, Cali, Colombia

[6] Programa de Medicina, Facultad de Ciencias de la Salud, Exactas y Naturales, Universidad Libre, Barranquilla 081007, Colombia

* Correspondence: carlosgrande@mail.uniatlantico.edu.co; dccarrascal@unibarranquilla.edu.co

Figure S1. Evaluation of the antimicrobial activity of NPs-SiO_2 against Gram-positive and Gram-negative bacterial strains.

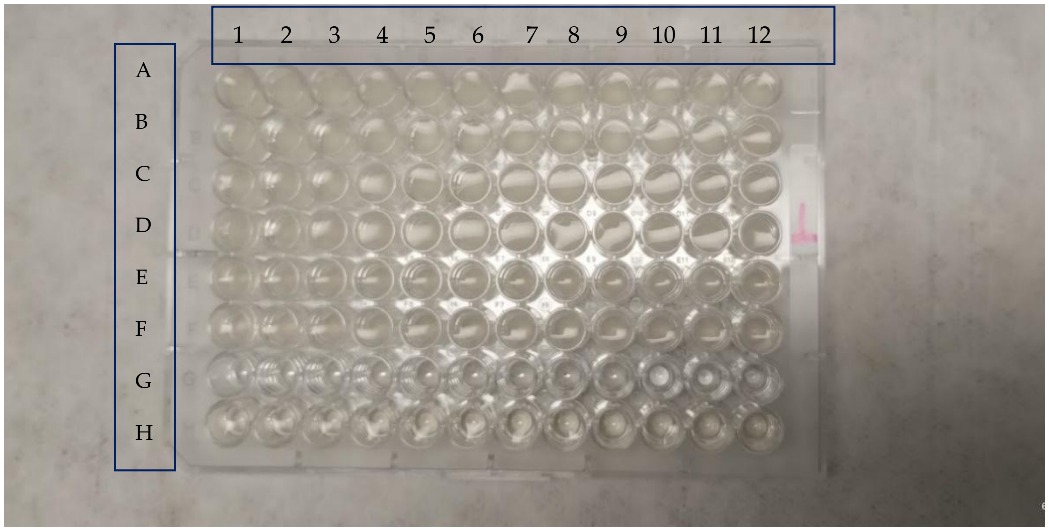

International Journal of
Molecular Sciences

MDPI

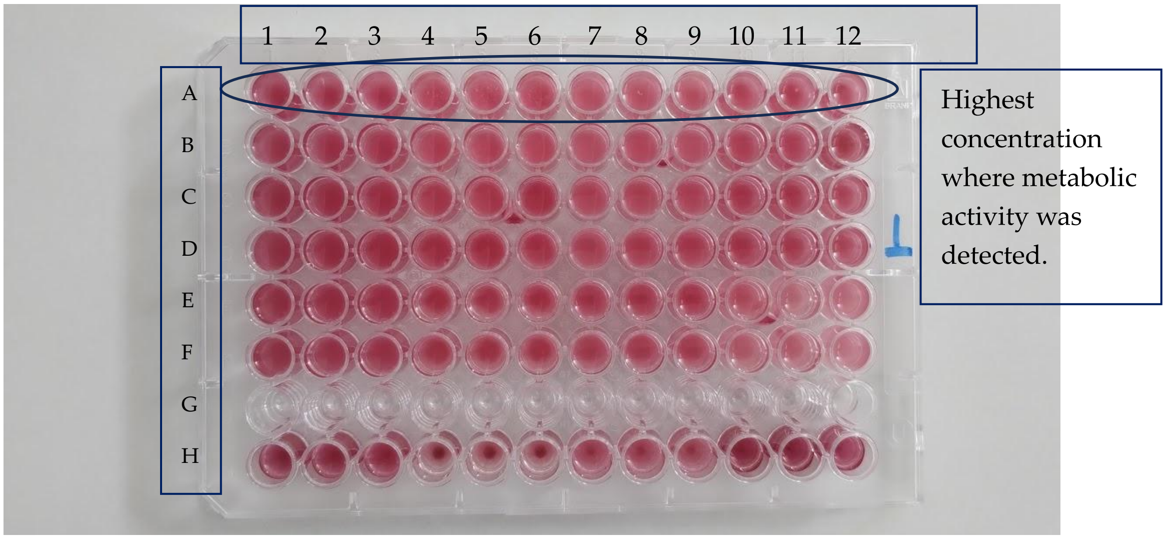

NPs-SiO2 (mg/L)	Horizontal line
60	A
30	B
15	C
7.5	D
3.75	E
1.88	F
Negative control	G
Positive control	H

Strain	Vertical line		
E. hormaechei	1	2	3
K. variicola	4	5	6
S. enterica	7	8	9
S. aureus	10	11	12

Figure S2. Evaluation of the antimicrobial activity of CUR against Gram-positive and Gram-negative bacterial strains.

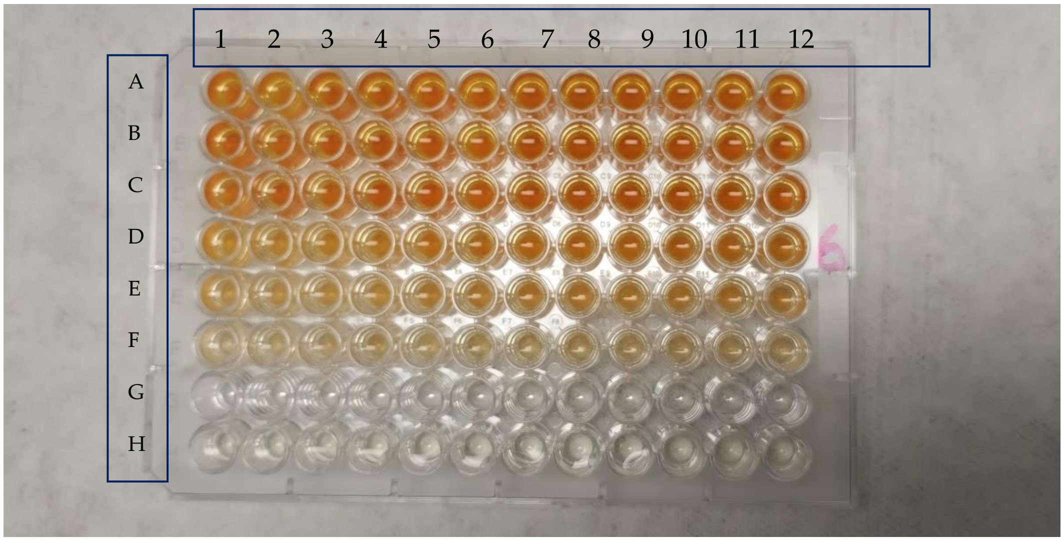

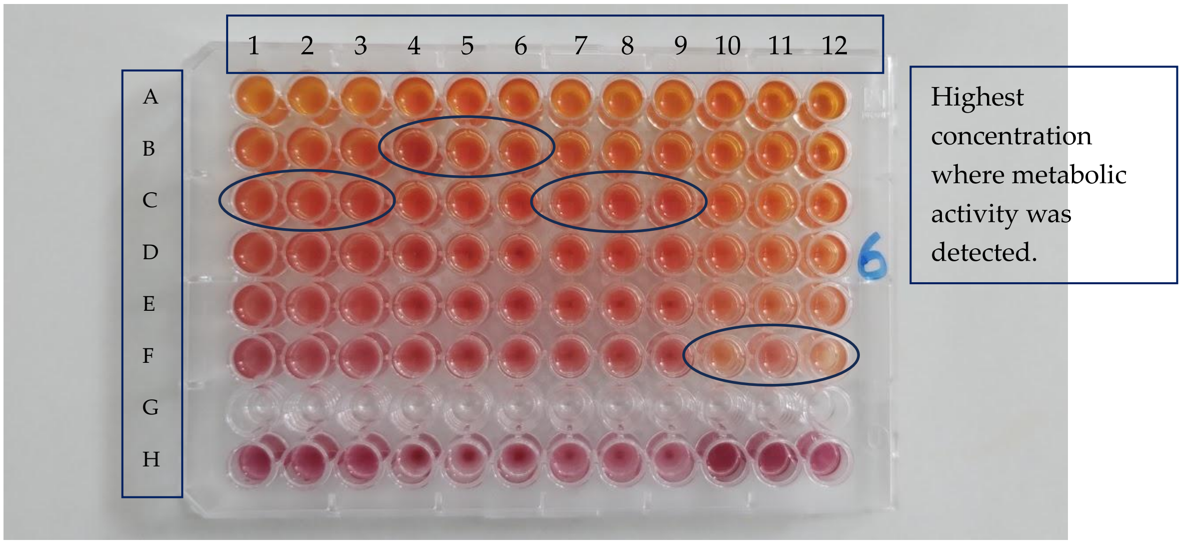

CUR (mg/L)	Horizontal line
30	A
15	B
7.5	C
3.75	D
1.88	E
0.94	F
Negative control	G
Positive control	H

Strain	Vertical line		
E. hormaechei	1	2	3
K. variicola	4	5	6
S. enterica	7	8	9
S. aureus	10	11	12

Figure S3. Evaluation of the Biofilm inhibition of NPs-SiO_2 against Gram-positive and Gram-negative bacterial strains.

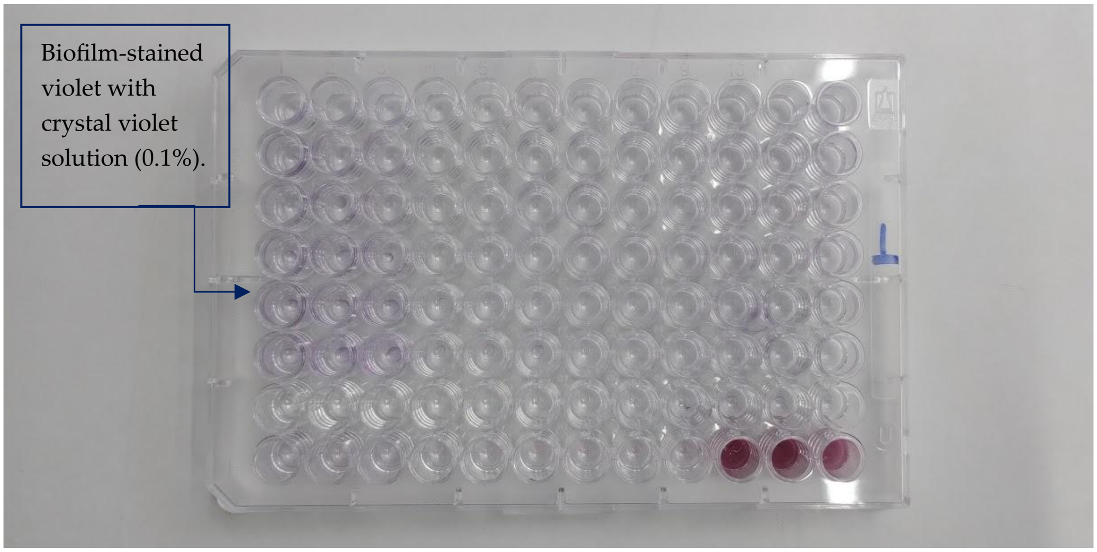

International Journal of
Molecular Sciences

MDPI

Figure S4. Evaluation of CUR biofilm inhibition against Gram-positive and Gram-negative bacterial strains.

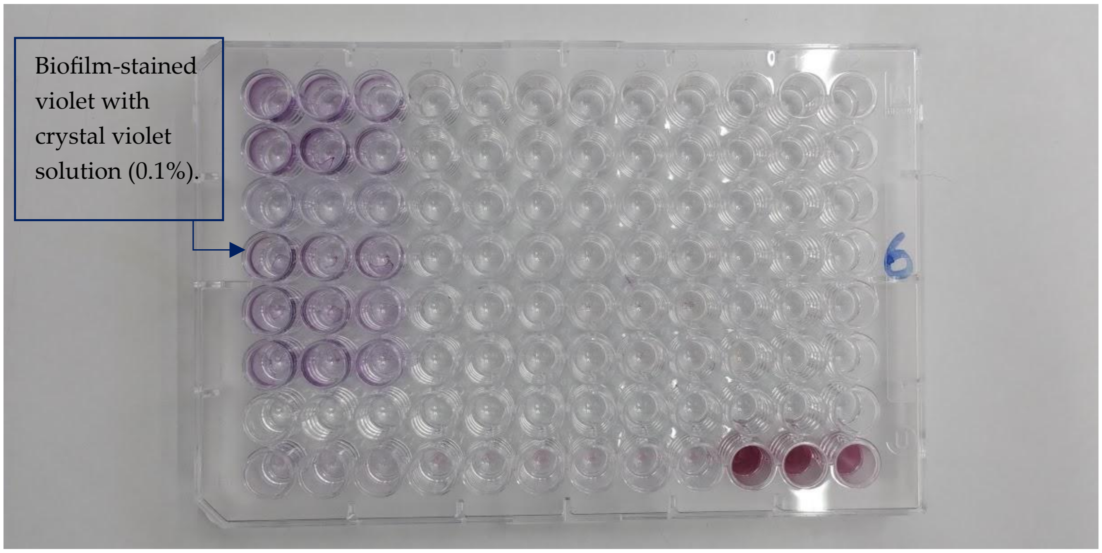

Supplement: Supplementary file 1 [file ijms-27-06856-s001.zip › ijms-4410233-supplementary.pdf]
